# Recent Advancements in Lung Cancer Metastasis Prevention Based on Nanostrategies

**DOI:** 10.1002/advs.202409293

**Published:** 2025-03-26

**Authors:** Fan Xu, Yao Liu, Zujun Que, Bin Luo, Yun Yang, Yan Li, Zhanxia Zhang, Jianhui Tian

**Affiliations:** ^1^ Department of Oncology, Shanghai Municipal Hospital of Traditional Chinese Medicine Shanghai University of Traditional Chinese Medicine 274 Zhijiang Road Shanghai 200071 China; ^2^ Research Center for Cancer Shanghai Municipal Hospital of Traditional Chinese Medicine Shanghai University of Traditional Chinese Medicine 274 Zhijiang Road Shanghai 200071 China; ^3^ Cancer Institute, Longhua Hospital Shanghai University of Traditional Chinese Medicine 725 Wanping South Road Shanghai 200032 China

**Keywords:** clinical translation, lung cancer, metastasis, nanodrug delivery systems, nanostrategies, the invasion‐metastasis cascade process

## Abstract

Metastasis is the leading cause of death in patients with lung cancer. Multidisciplinary comprehensive treatments (MDT), including surgery, chemotherapy, radiotherapy, gene‐targeted therapy, immunotherapy, antibody‐drug conjugate (ADC), natural products, etc., have been currently used for lung cancer metastasis. The MDT model has shown promising efficacy against lung cancer metastasis in clinical practice. However, these therapies have some limitations, such as unusual toxic side effects, drug resistance, limited indications, and high costs. Therefore, emerging technological platforms are imperative to overcome these bottlenecks. Nanomedicine can be used to prepare efficient drug delivery systems owing to its good biocompatibility, high targeting, responsive release, and multidrug codelivery and plays an important role in the synergistic antimetastasis of lung cancer because of the optical, acoustic, electrical, thermal, and magnetic functions. This comprehensive review analyses the limitations of the MDT model, briefly outlines the advantages of nanotechnology, introduces the promising nanodrug delivery systems, summarizes the emerging nanostrategies against lung cancer metastasis based on the invasion‐metastasis cascade process, and provides a summary of the prospects and challenges for the clinical translation of nanomedicines.

## Graphical abstract

Metastasis is the leading cause of death in patients with lung cancer. Nanomedicine can be used to prepare efficient drug delivery systems owing to their advantages and plays an important role in the synergistic antimetastasis of lung cancer. This comprehensive review summarizes the emerging nanostrategies against lung cancer metastasis based on the invasion‐metastasis cascade process.

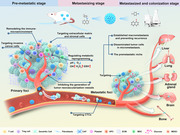

## 1 Introduction

According to the latest statistics released by the International Agency for Research on Cancer, lung cancer is the most fatalmalignant tumor with the highest mortality rate globally.^[^ ^1^ ^]^ The 5‐year survival rate of lung cancer is lower than 15%,^[^ ^2^ ^]^ among which 90% of deaths are due to metastasis rather than the primary tumor. The management of lung cancer metastasis is considerably more difficult than that of primary lung cancer. Lung cancer metastasis is a complex, multistep pathological process termed the invasion‐metastasis cascade.^[^ ^3^, ^4^, ^5^ ^]^ The process of invasion‐metastasis cascade in lung cancer can be broadly summarized in three stages: 1) premetastatic stage: Cancer cells in the primary focus undergo epithelial‐mesenchymal transition under the stimulation of some factors and obtain the characteristics of mesenchymal cells (such as enhanced migration and invasion) by losing polarity and intercellular connection.^[^ ^6^, ^7^, ^8^ ^]^ Next, these invasive cancer cells degrade the extracellular matrix (ECM) by secreting cytokines, such as Matrix Metalloproteinase‐2 and Matrix Metalloproteinase‐9 (MMP‐9), and gradually migrate to blood vessels under the guidance of vascular chemokines. Then they pass through the vascular endothelium and into the hematologic system (intravasation),^[^ ^9^ ^]^ 2) metastasizing stage: There is an abundant blood supply in the lungs, which enables cancer cells that have passed through the ECM to spread all over the body like “seeds” in the blood and stagnate in distant tissues and organs; 3) metastasized and colonization stage: Cancer cells extravasate and, and survive in the local immune response of these target organs' microenvironment (soil), forming potential micrometastasis.^[^ ^10^, ^11^, ^12^ ^]^ These cancer cells have the characteristic of long‐term dormancy and can be hidden in the body for several years.^[^ ^13^, ^14^, ^15^ ^]^ Once stimulated by proliferation‐inducing signals and angiogenesis factors, they will quickly activate and proliferate and eventually develop into large‐scale metastasis.^[^ ^16^ ^]^

Consequently, lung cancer metastasis occurs long before the formation of macroscopic metastasis, potentially delaying the diagnosis and treatment of metastasis. Currently, MDT model, including surgery, chemotherapy, radiotherapy, gene‐targeted therapy, immunotherapy, antibody‐drug conjugate (ADC), natural products, etc., is used for lung cancer metastasis with the aim of improving treatment outcomes and patient survival. However, there are many challenges and bottlenecks in this process, such as the treatment of suitable population, drug resistance, and side effects, which greatly hinder its clinical application. **Table** **1** details the suitable population, recent advancements, advantages, and disadvantages of MDT model.

**Table 1:** Control status of lung cancer metastasis: the MDT model for lung cancer.

Types	The suitable population	Recent advancements	Advantages	Disadvantages
Surgery	As a local treatment modality, surgery serves as the primary radical therapy for Stage I – III lung cancer. It can also be used in some special circumstances in Stage IV patients (e.g., oligo‐metastasis, palliative treatment).	Minimally invasive surgery; optimization of the extent of surgical resection; individualized surgical protocols; imaging techniques, such as 3D reconstruction techniques and intraoperative navigation techniques.	The basic clinical principle is still to actively pursue surgical treatment, which can directly remove the tumor tissue, eliminate, or reduce the tumor load, improve the symptoms and achieve long‐term survival.	Limited scope of application, only suitable for early to mid‐stage patients, limited efficacy in the treatment of advanced tumors; surgical risk; risk of the presence of residual foci; surgical complications; lack of antimetastatic specificity.
Radiotherapy	For patients with stages I‐IV, and can be used for the local treatment of inoperable lung cancer patients	Precision of radiotherapy coverage; diversification of radiotherapy forms; diversification of radiotherapy equipment.	Precise striking of lesions; noninvasive treatment: suitable for patients in poor physical condition who cannot tolerate surgery; wide range of applications; relatively controllable adverse effects.	Local adverse effects, such as skin lesions, radioinflammation; systemic adverse effects, such as myelosuppression, gastrointestinal reactions; relatively limited efficacy in advanced patients; lack of antimetastatic specificity.
Chemotherapy	Typically used for stage IB‐IV lung cancer, either after surgery for early to mid‐stage lung cancer or advanced lung cancer with distant metastasis; meeting stringent chemotherapy criteria	Predominantly platinum‐containing regimens; diversified forms of chemotherapy; combination therapy.	Wide scope of application; can reach the whole body with blood circulation, effectively removing residual cancer cells in the body; diversity of drug types and protocols to achieve individualized treatment.	High systemic toxic reactions; chemotherapy resistance; individual differences affecting efficacy; lack of tumor‐specific targets; severe malnutrition, cachexia, severe impairment of organ function, severe infections, and other conditions cannot be used.
Gene‐targeted therapy	Lung cancer patients who are intolerant to chemotherapy and have mutations in specific driver genes	Discovery and drug development of new lung cancer‐related targets: EGFR, ALK, ROS1, RET, MET, BRAF inhibitors, etc.	High precision; antimetastatic specificity; significant efficacy; easy oral administration.	Drug‐resistant; limited targets; expensive.
Immunotherapy	Patients with early, locally advanced and advanced lung cancer with high tumor mutational burden or high expression of PD‐L1, as well as patients with specific genetic mutations, have been covered.	Novel immunotherapeutic drug development: PD – 1/L1 inhibitors, CTLA‐4 inhibitors; development of new combinations: immunotherapy in combination with chemotherapy, targeted therapy, radiotherapy, and other modalities; optimization of biomarkers; expansion of therapeutic scope: has been entered perioperative treatment.	Durable antitumor response; relatively mild side effects; effective inhibition of micrometastasis	Drug resistance; risk of irAEs; lack of precise predictors; expensive; risk of hyper‐progression; long‐term effects on the immune system: autoimmune disorders, risk of immune depletion, etc.
ADC	Advanced patients with disease progression despite conventional chemotherapy, targeted therapy, and immunotherapy, especially those with distant metastasis;Positive expression of specific targets	New drug development and approval: trastuzumab‐delutecan (DS‐8201); combination therapy exploration; expanding therapeutic reach	Precision‐targeted therapy; potent antitumor activity; diverse and individualized treatment.	Adverse reactions; resistance problems; high cost of drugs.
Natural products	Suitable for a wide range of people, and can be adapted to patients with early, middle and late stage lung cancer	Novel active ingredient discovery; drug dosage form improvement; combination applications with traditional therapies; combination studies with emerging therapies	Remarkable antitumor efficacy; low toxicity and side effects; mitigation of other toxic side effects; natural source, relatively low side effects; multitarget, holistic regulatory effects; rich resources and potential drug diversity	Problems of active ingredient content and stability; complex drug action mechanisms and research difficulties; lack of standardization and normalization.

**Figure 1:**
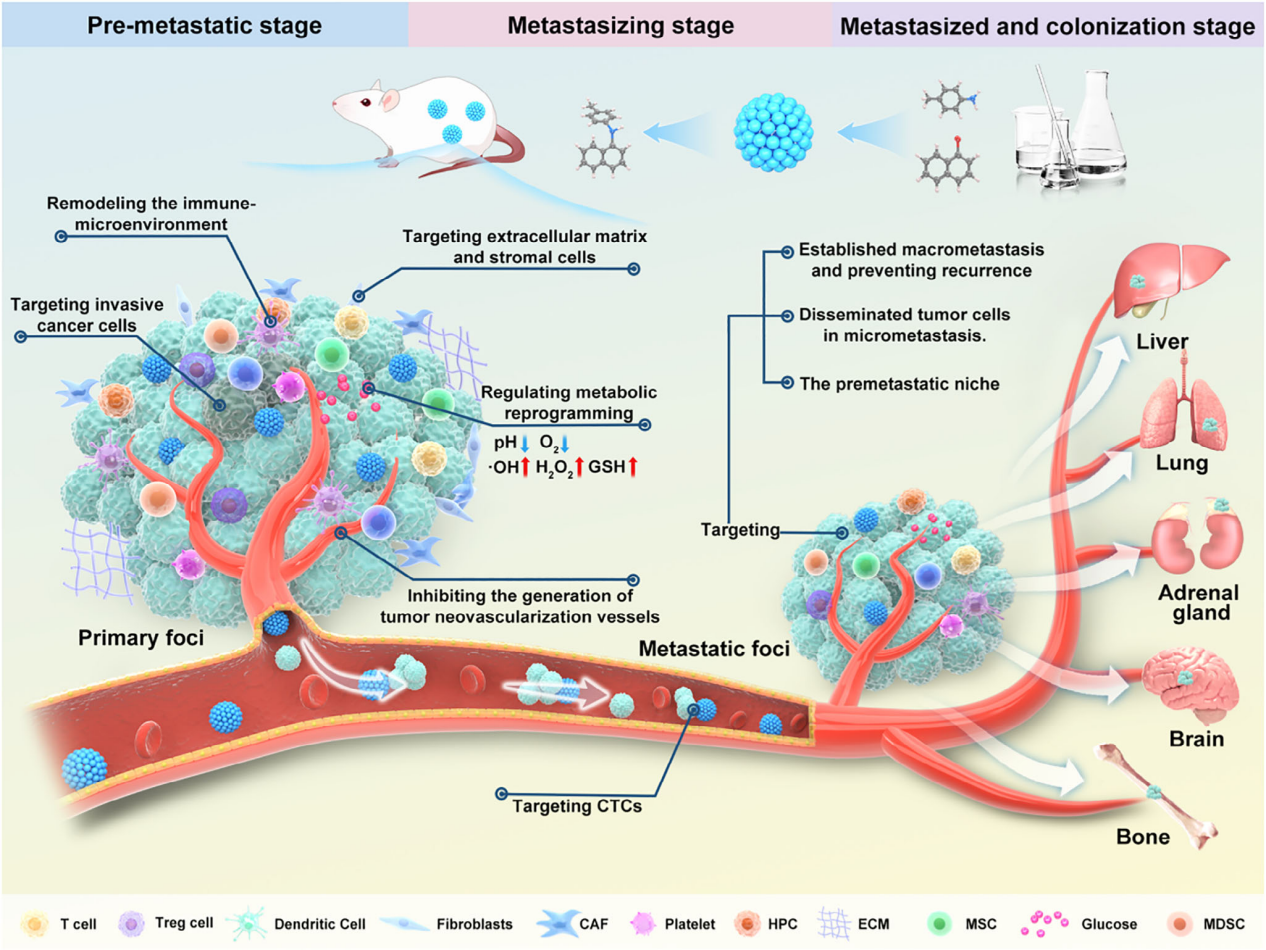
Overview of nanostrategies in the prevention of lung cancer metastasis based on the invasion‐metastasis cascade process.

Therefore, emerging technologies and strategies are required to overcome lung cancer metastasis and achieve effective clinical translation. Recently, nanotechnology has received considerable attention in medicine, has become an emerging wave in medical research, and has opened a new area of research with advancement and interdisciplinarity‐the field of nanomedicine.^[^ ^17^, ^18^, ^19^ ^]^ In the field of nanomedicine, a series of medical materials, including nano drug delivery systems (NDDS),^[^ ^20^, ^21^, ^22^ ^]^ nano drugs,^[^ ^23^ ^]^ and nano organs,^[^ ^24^, ^25^ ^]^ are created by studying nanomaterials and their properties (physical, chemical, biological, mechanical, and electrical) at the nanoscale (1–1000 nm). These materials are widely used in clinical diagnosis, treatment, and medical research. Many antimetastasis nanostrategies have been developed in recent years, with considerable progress. Therefore, this review focused on the prevention and treatment of lung cancer metastasis, outlined the advantages of nanotechnology and promising NDDS, and summarized recent nanostrategies for lung cancer metastasis based on the invasion‐metastasis cascade process (**Figure** **1**). Finally, the main challenges and future developments in nanotechnology against lung cancer metastasis are summarized to stimulate more innovative research on designing nanoplatforms with better therapeutic efficacy and simplified components.

## 2 Advantages of Nanotechnology Against Lung Cancer Metastasis

### 2.1 Multidrug Codelivery

Tumors are highly heterogeneous, as evidenced by the heterogeneity between tumors in different patients (inter‐tumor heterogeneity) or between tumor cells within a single tumor (intra‐tumor heterogeneity).^[^ ^26^, ^27^, ^28^ ^]^ Therefore, monotherapy does not achieve the desired therapeutic effect in the treatment of lung cancer metastasis. The current intervention strategy for lung cancer metastasis is based on the MDT model. The MDT model is based on the patient's physical condition, pathological histological type, molecular typing of the tumor, extent of invasion, and developmental trend. This is followed by the planned combination use of various measures, such as surgery, radiotherapy, chemotherapy, targeted molecular therapy, immunotherapy, ADC, and natural products. Compared with monotherapy, MDT could be more effective in inhibiting lung cancer metastasis through multiple mechanisms, resulting in synergistic effects and reduced toxicity to maximize resistance to lung cancer metastasis.^[^ ^29^, ^30^, ^31^, ^32^ ^]^

**Figure 2:**
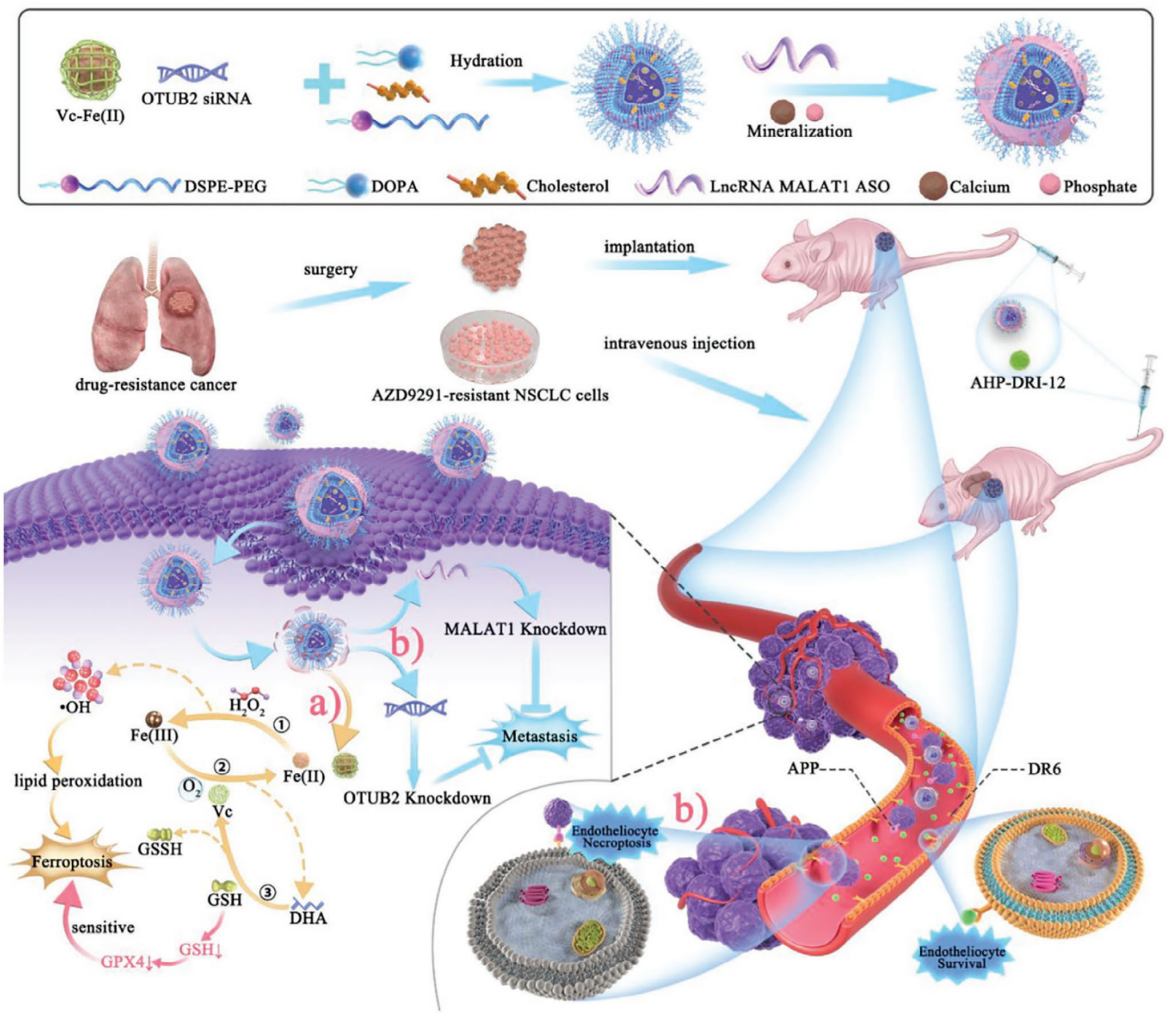
Schematic illustration of tumoricidal effect of combination therapy of VF/S/A@CaP and AHP‐DRI‐12. Reproduced with permission.^[^ ^33^ ^]^ Copyright 2022, Wiley.

Nanotechnology allows the codelivery of two or more drugs that act synergistically on multiple pathways and targets simultaneously. For example, AZD9291 is a third‐generation epidermal growth factor receptor tyrosine kinase inhibitor (EGFR‐TKI) used for the prevention of recurrent metastasis of nonsmall cell lung cancer (NSCLC). However, patients often develop resistance to AZD9291 after using it alone. Wang's team study aimed to overcome AZD9291 resistance through iron death (ferroptosis) and multitarget interference.^[^ ^33^ ^]^ A nanocatalytic sensitizer (VF/S/A@CaP) containing vitamin C (Vc), Fe(II), si‐OTUB2 (siRNA targeting OTUB2), and ASO‐MALAT1 (antisense oligonucleotide targeting long‐stranded noncoding RNA MALAT1) was designed for the study. Vc‐Fe(II) was generated by Fenton reaction to produce hydroxyl radical (*─*OH), which induces iron death. Meanwhile, the oxidation product of Vc, consumed glutathione (GSH), which further inhibited glutathione peroxidase 4 and enhanced iron death. Dual‐targeted knockdown of si‐OTUB2 and ASO‐MALAT1 inhibited the metastatic ability of tumor cells. The study also used AHP‐DRI‐12, a peptide inhibitor that inhibits extravasation of tumor cells through the vascular wall, which further inhibited hematogenous metastasis. In vitro experiments showed that AZD9291‐resistant NSCLC cells were more sensitive to Vc‐Fe(II)‐induced iron death and that Vc‐Fe(II)@CaP significantly inhibited tumor cell growth and metastasis. In in vivo experiments, VF/S/A@CaP significantly inhibited tumor growth and metastasis in AZD9291‐resistant NSCLC, especially in a patient‐derived xenograft model, with a tumor inhibition rate of 91.39%. Treatment in combination with AHP‐DRI‐12 further enhanced the antimetastatic effect. This combination therapy strategy demonstrated significant antitumor effects both in vivo and ex vivo, providing a new therapeutic idea to overcome AZD9291‐resistant NSCLC (**Figure** **2**).

### 2.2 Targeting

Owing to the potential toxicity of antimetastatic drugs to healthy tissues, the precise delivery of drugs to tumor sites is one of the biggest challenges in their clinical application. Nanotechnology enable precise delivery of drugs to targeted sites. Targeting strategies for NDDS mainly include passive, active, physical, and other types of targeting.

#### 2.2.1 Passive Targeting

In the tumor microenvironment (TME), malignant, and stromal cells secrete various angiogenic factors that induce neovascularization and vascular remodeling. The tumor vasculature is highly disordered, with tortuous tumor vessels and functional abnormalities leading to increased permeability. In addition, compromised lymphatic drainage can result in lymphatic obstruction. Interstitial pressure at the center of the tumor was higher than that at the periphery. These features allow the nanoparticles (NPs) to selectively accumulate and remain in the tumor interstitium, leading to an enhanced permeability and retention (EPR) effect. In solid tumors, the passive targeting of NPs relies on the EPR effect, which allows nanomedicines to more easily leak from the tumor vasculature and accumulate at the tumor site than those of nonencapsulated drugs.^[^ ^34^, ^35^, ^36^ ^]^

#### 2.2.2 Active Targeting

Passive targeting, mediated by the EPR effect, enables drug enrichment in solid tumors. Although the EPR effect of nanomedicines has been demonstrated in preclinical xenograft mouse models, clinical translation remains challenging owing to the dense ECM, high interstitial fluid pressure levels, tumor heterogeneity and complexity, and other factors.^[^ ^37^, ^38^ ^]^ Active targeting strategies, which rely on ligand–receptor recognition patterns, have been proposed to overcome the limitations of passive targeting. Some specific receptors are overexpressed on the surface of lung cancer cells but are hardly or less expressed in normal cells. Biorecognition molecules are attached to the surface of NPs to target specific receptors that are overexpressed by neoplastic cells. Receptor‐mediated endocytosis improves tumor targeting and drug accumulation. The most researched targeting ligands currently include small molecules, such as folic acid and glycans; large molecules such as peptides, proteins, antibodies, aptamers, and oligonucleotides; and various biomimetic targeting materials, such as cell membranes and bacteria that have been developed in recent years.^[^ ^39^, ^40^, ^41^ ^]^

**Figure 3:**
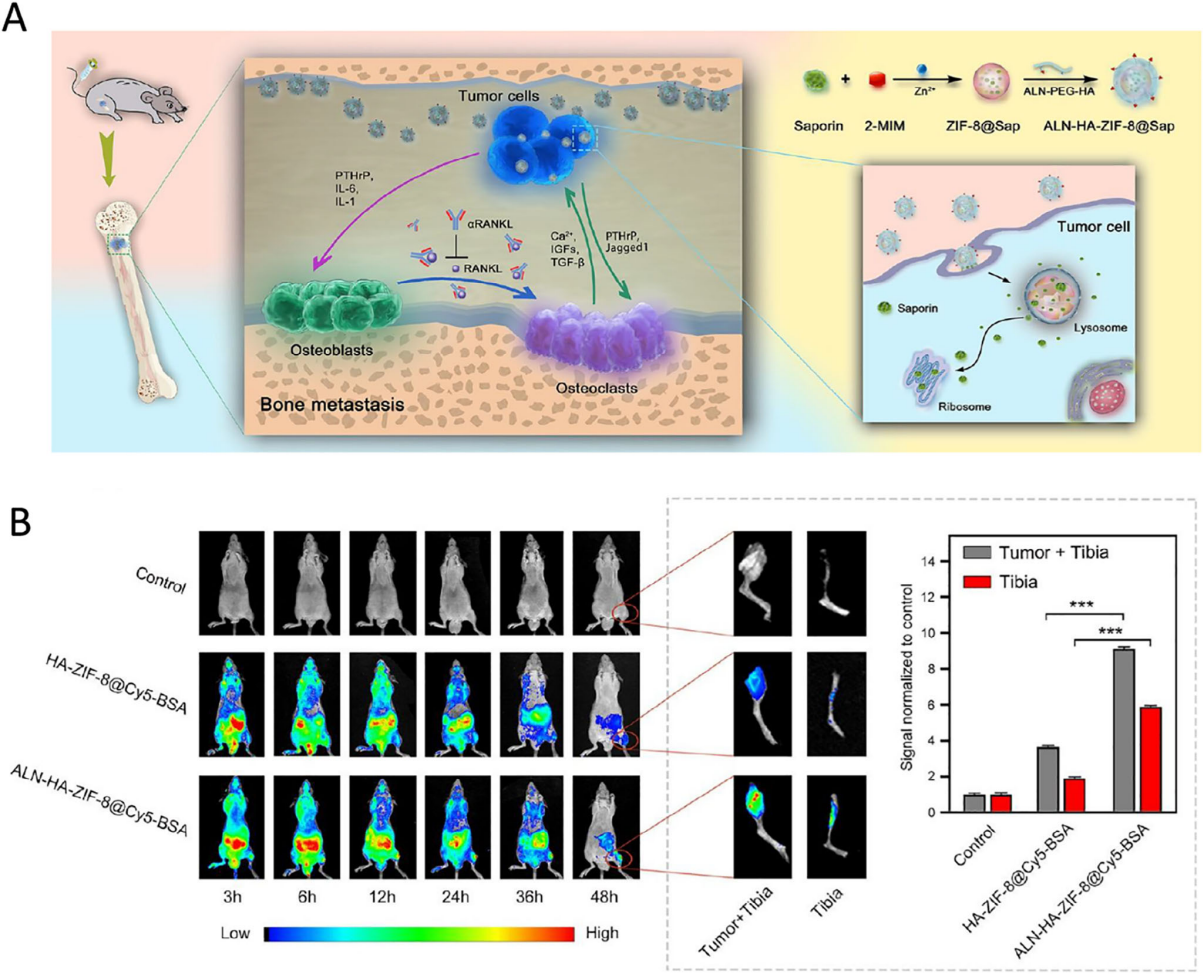
Applications of nanomedicine in active targeting. A) The schematic representation of bone‐seeking and CD44‐receptor‐targeting biomineralized metal–organic framework (MOF) nanoparticles ALN‐HA‐ZIF‐8@Sap nanoparticles, in combination with RANKL antibody inhibit bone metastasis and prevent skeletal‐related events. B) In vivo distribution of bone‐tumor‐bearing mice intravenously injected with Cy5‐labeled NPs at 3, 6, 12, 24, 36, and 48 h after administration, respectively. *p < 0.05, **p < 0.01, and ***p < 0.001 analyzed by Student's *t*‐test, one‐tailed. Reproduced with permission.^[^ ^42^ ^]^ Copyright 2022, American Chemical Society.

As shown in **Figure** **3**, Niu et al. developed a nanomedicine (ALN‐HA‐ZIF‐8@Sap) around lung cancer bone metastasis. It is well known that bone metastasis is a common metastatic site of lung cancer and is often accompanied by bone loss, fracture, and other SREs. The interactions between tumor cells and bone cells (e.g., osteoblasts and osteoclasts) in the bone microenvironment form a vicious circle that promotes tumor proliferation and bone destruction. Existing therapeutic strategies have focused on inhibiting tumor cell proliferation or blocking osteoclast activation, but few studies have intervened in both. The researchers developed this dual‐protein therapeutic nanomedicine to simultaneously inhibit tumor cell proliferation and osteoclast activation, intervening in the vicious cycle between the bone microenvironment and tumor cells. In this, achieving dual targeting of bone and tumor cells is the focus. The researchers modified alendronic acid (ALN) and hyaluronic acid (HA) on the surface of the nanomedicine. The results confirmed that the nanomedicine had good bone targeting and tumor cell targeting properties. The combination of ALN‐HA‐ZIF‐8@Sap with RANKL antibody significantly inhibited bone metastasis and reduced SREs, such as bone loss. The mice in the combination treatment group had significantly prolonged survival and better preservation of bone structure.^[^ ^42^ ^]^ Spinal metastasis patients have a low therapeutic response rate to conventional immune checkpoint inhibitors, such as anti‐PD‐1/PD‐L1 antibodies. Libo Jiang's team prepared RGDyK‐modified, zinc protoporphyrin (ZnPP)‐loaded mesoporous silica NPs (ZnPP@MSN‐RGDyK), known as Z@M‐R. Among them, RGDyK serves as a targeting peptide that can precisely target the NSCLC spinal metastasis and enhance its immunotherapy efficacy.^[^ ^43^ ^]^

#### 2.2.3 Physicochemical Targeting

Physicochemical targeting strategies use the unique electrical, thermal, and magnetic effects of nanomaterials themselves, induced by physicochemical conditions, such as a magnetic field and temperature, to enrich antitumor drugs at the tumor site. The physicochemical factors currently studied are divided into two categories: endogenous factors, which are mainly derived from the pathophysiological environment specific to the tumor tissue, such as pH, redox potential, enzymes, oxygen content, ionic strength, and shear force; and exogenous factors, which mainly include physical stimuli, such as magnetism,^[^ ^44^, ^45^ ^]^ heat, light, sound, and electricity.

Wang et al. utilized a laser‐integrated magnetic drive system. This system actively transported magnetic nanorobots embedded with DOX to the tumor site for localized hyperthermia and chemotherapy. Inside the lumen, through the motion controlled by a torque‐force hybrid magnetic field, these magnetic nanorobots aggregated at a fixed point corresponding to the position of the positioning laser. They were able to overcome gravity and move upward over long distances. Moreover, under the guidance of ultrasound imaging, they were enriched at the tumor site. The release of DOX at the tumor site was accomplished by the photothermal effect induced by near‐infrared laser irradiation. The magnetic‐field‐driven targeting ability enhanced the photothermal treatment of nanomedicines, while simultaneously minimizing damage to off‐target tissues. This approach offers a potentially clinically viable strategy for tumor‐targeted drug delivery.^[^ ^46^ ^]^

#### 2.2.4 Other Targeting

**Figure 4:**
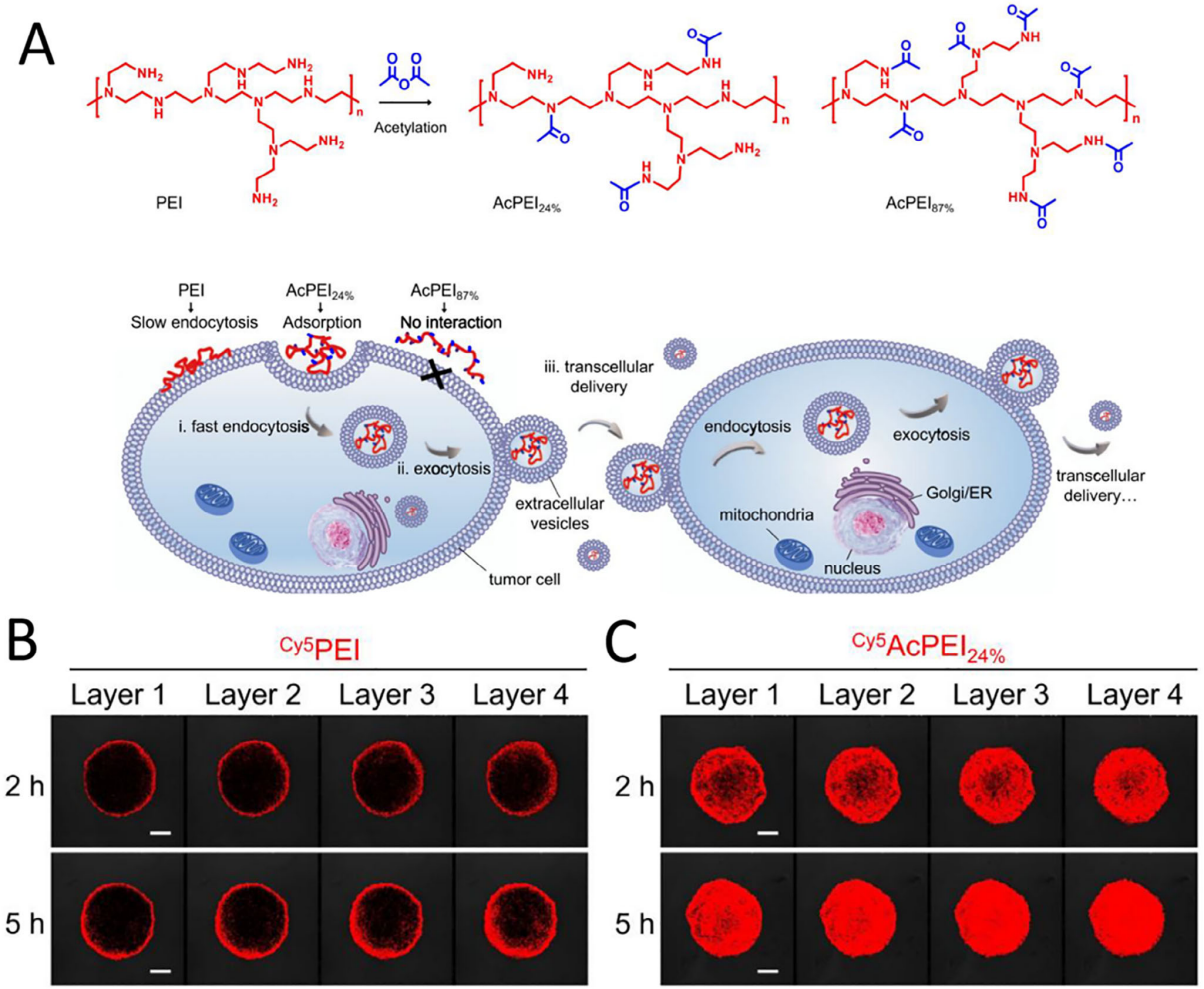
Transcytosis of polyethylenimine (PEI) mediates targeting of nano drugs. A) Different intracellular pathways of AcPEIs with different acetylation degrees in tumor cells. B,C) Penetration ability of Cy5AcPEIs in tumor spheroids. Reproduced with permission.^[^ ^49^ ^]^ Copyright 2021, American Chemical Society.

In addition, in recent years researchers have discovered other nanotargeting mechanisms,^[^ ^47^, ^48^ ^]^ such as adsorption‐based mediated transcytosis (AMT) is a novel paradigm. It triggers the binding of nanomedicines to negatively charged cell membranes through electrostatic interactions, enabling active transcytosis and tumor cell‐to‐cell delivery. It can overcome multiple biological barriers to achieve effective drug penetration and accumulation in tumors. For example, some researchers have explored the effect of the charge density of cationic polymers, such as polyethyleneimine (PEI) on their AMT‐induced tumor penetration ability. They obtained acetylated PEIs (AcPEIs) with different cationic charge densities and assessed their tumor penetration efficiency using an in vitro multilayer tumor spheroid model. The experimental results showed that the cytotoxicity, zeta potential, and cell‐binding affinity of AcPEIs were significantly reduced with increasing degree of acetylation. Highly acetylated AcPEIs (e.g., AcPEI 87%) were inefficient in endocytosis and transcytosis due to their weak cell‐binding ability, whereas unacetylated PEIs were equally ineffective in inducing transcytosis despite their strong cell‐binding ability. AcPEI with 24% acetylation (AcPEI 24%) showed the highest transcytosis efficiency, and its moderate cell‐binding affinity triggered rapid adsorption‐mediated endocytosis, followed by efficient transcytosis via the Golgi and endoplasmic reticulum‐mediated exocytosis pathways. It was shown that AMT is another new mode to improve nanoparticle targeting. By adjusting the charge density of cationic polymers, an effective way to optimize their AMT‐induced tumor penetration efficiency and targeting can be achieved (**Figure** **4**).^[^ ^49^ ^]^

### 2.3 Controlled Release

The controlled release capability of drugs plays a crucial role in tumor therapy. By precisely controlling the release rate and release time of the drug, the drug can reach an effective therapeutic concentration at the tumor site while avoiding damage to healthy tissues. Stimuli‐responsive nanomaterials can be used to prepare controlled‐release delivery systems. Through its precise identification of tumor tissues and cells and targeted release mechanism, the NDDS not only improves the therapeutic effect of the drugs and makes the drugs more enriched in the tumor tissues, but also effectively reduces the toxicity and side‐effects of the drugs on the normal tissues, thus providing a safer and more effective means for tumor treatment. The most representative of these are the nanodrugs based on the TME response. Compared with normal tissues, the TME exhibits rapid proliferation and metabolism, with an acidic pH, high expression of hydrogen peroxide (H_2_O_2_), high levels of enzymes and GSH, and severe hypoxia. Drugs transported via TME‐responsive NDDS can achieve efficient release of anticancer drugs under the influence of the acidic pH of TME, GSH, or specific enzyme catalysis.^[^ ^50^, ^51^, ^52^, ^53^, ^54^, ^55^, ^56^ ^]^

**Figure 5:**
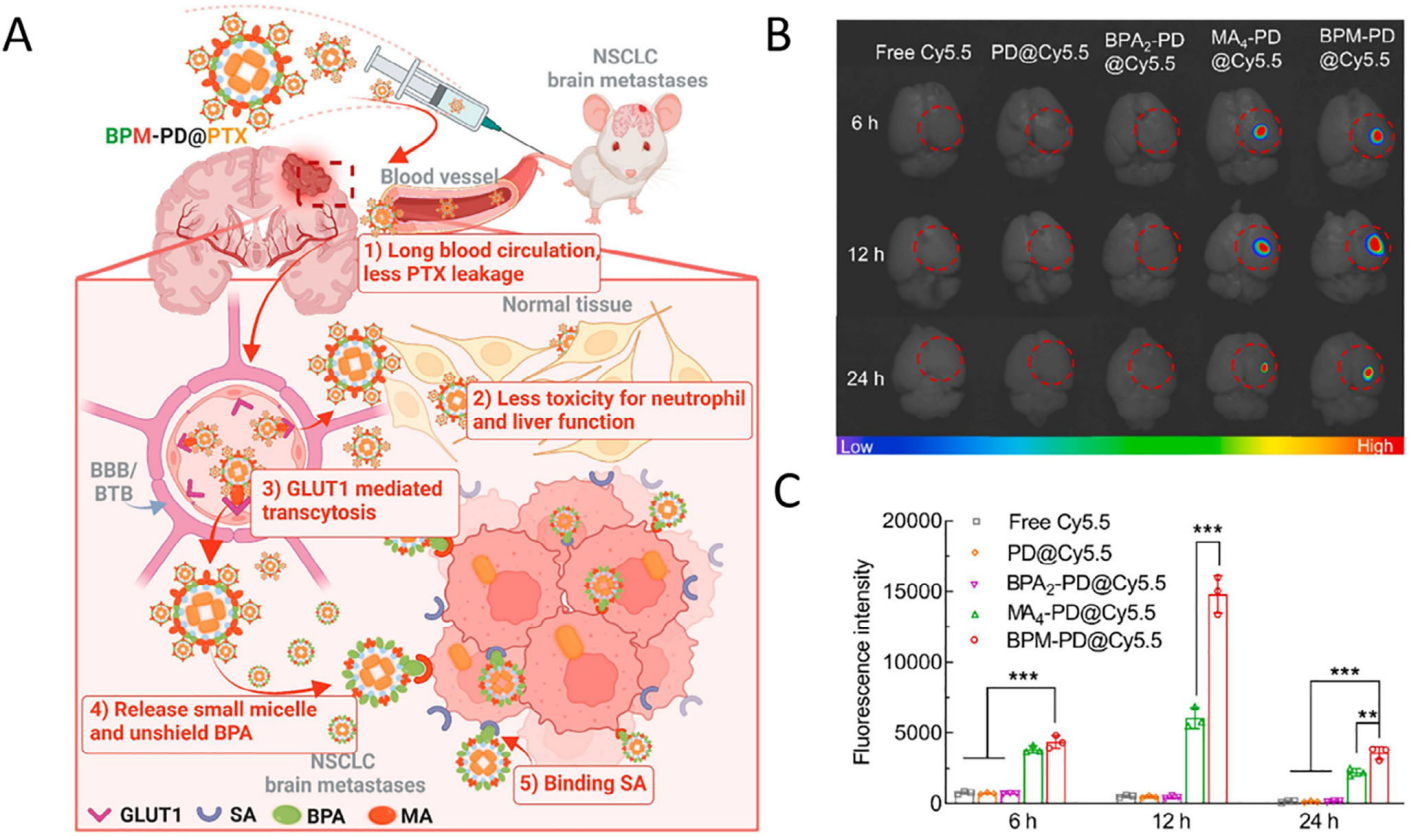
Applications of nanomedicine in controlled release property. A) Schematic representation of BPM‐PD, a unique “on/off” switchable release nanoformulation responsive to the acidic extra‐tumoral microenvironment. Ex vivo brain fluorescence images B) and quantitative analysis C) at various time points (6, 12, and 24 h) after intracranial injection of the free Cy5.5, PD@Cy5.5, BPA2‐PD@Cy5.5, MA4‐PD@Cy5.5, and BPM‐PD@Cy5.5 (*n* = 3). Reproduced with permission.^[^ ^59^ ^]^ Copyright 2024, Elsevier Sci. Ltd.

Zhang et al. developed charge‐reversal biodegradable mesoporous silica carrying the glycolysis inhibitor lonidamine and small interfering RNA against glutaminase dual‐mechanism‐based nutrient partitioning nanomodulator (DMNPN), which is a GSH‐ and pH‐sensitive nanoscale drug delivery system. In the current study, DMNPN responded to acidic pH and high concentrations of GSH, resulting in satisfactory drug release. Compared with free drugs, DMNPN significantly inhibits the growth and recurrent metastasis of tumors and enhances immunotherapy for PD‐1‐resistant tumors.^[^ ^57^ ^]^ A novel V‐based nanoplatform, pLi‐hollow vanadium‐doped mesoporous silica NPs (HVMSN)‐Pt, was designed by Ran et al. Platelet‐derived growth factor receptor‐β (PDGFR‐β) recognition cyclic peptide (PDGFB)‐labeled liposomes were coated on HVMSN loaded with a Pt(IV) prodrug. The nanoplatform actively targeted the tumor tissue and effectively responded to the weak acidity and high GSH of the TME, enabling precise delivery and intelligent release of the Pt(IV) prodrug and vanadium ions. The nanoplatform pLi‐HVMSN‐Pt can actively recognize A549 tumors, rapidly respond to the TME, and ultimately release the drug to effectively inhibit lung cancer growth and metastasis.^[^ ^58^ ^]^ Li et al. developed an “on/off” switchable cross‐linked paclitaxel (PTX) nanocarrier, BPM‐PD. The carrier has a novel ultra pH‐sensitive junction (pH 6.8–6.5), and BPM‐PD exhibits a unique “on/off” switchable release of the anticancer drug PTX in response to the acidic extra‐tumoral microenvironment (**Figure** **5**). The “off” state of BPM‐PD@PTX effectively prevents premature release of the drug in the circulation, BBB/blood‐tumor barrier (BTB), and normal brain tissue, which is superior to that of the clinical PTX nanoformulation (NAB‐PTX). Moreover, the “on” state facilitates precise delivery to NSCLC brain metastatic cells. Compared with NAB‐PTX, BPM‐PD@PTX showed higher therapeutic efficacy, reduced tumor area (only 14.6%), prolonged survival time, and attenuated the adverse effects of aspartate aminotransferase and alanine aminotransferase (more than 83.7%), providing a promising approach for the treatment of brain metastasis of NSCLC.^[^ ^59^ ^]^

### 2.4 Synergistic Antimetastasis

The nanoscale dimensions offer unique physical, chemical, and biological properties to NPs, such as small size effect, high specific surface area, quantum size effect, macroquantum tunneling effects. They are distinguished from conventional materials which impart optical, acoustic, electrical, thermal, magnetic, and enzymatic properties to nanomedicines, which make them promising in the field of antidiversion.^[^ ^60^, ^61^, ^62^, ^63^, ^64^, ^65^, ^66^, ^67^, ^68^, ^69^ ^]^ The therapeutic effects of these nanomaterials may synergize with those of drugs to enhance anticancer efficacy.

**Figure 6:**
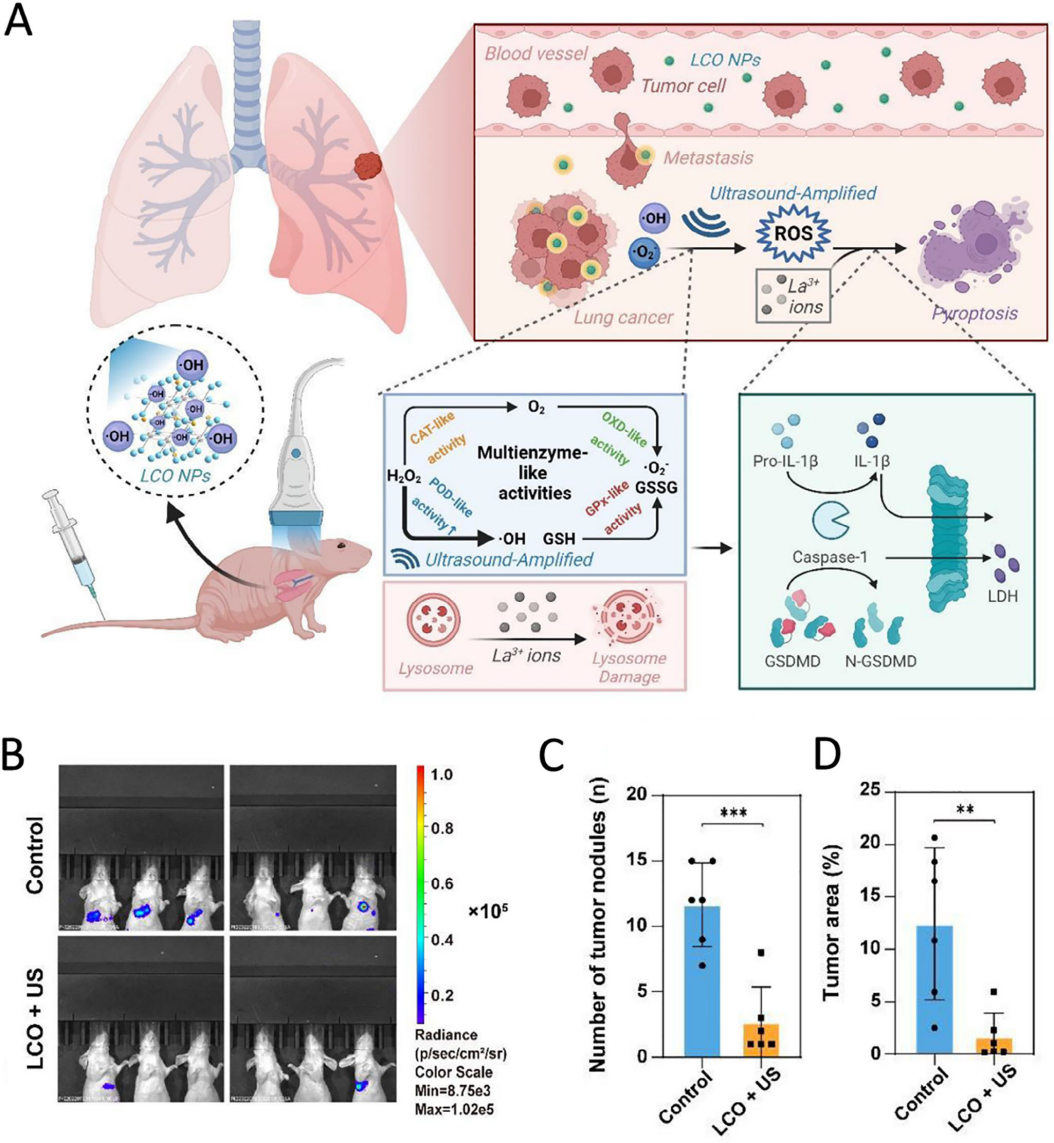
Applications of nanomedicine in synergistic anti‐metastasis. A) LaCoO_3_ nanocrystals with multienzyme properties synergize against lung cancer metastasis. B) In vivo bioluminescence images monitoring thoracic metastasis of H838‐luc cells after LCO nanoparticles and ultrasound (US) irradiation treatment (*n* = 6). Comparison of C) nodule number and D) area (%) of visible tumor nodules at representative images of hematoxylin and eosin‐stained lung tissues between the control and LCO + US groups. Reproduced with permission.^[^ ^70^ ^]^ Copyright 2023, Wiley‐VCH.

Researchers have taken advantage of the enzyme‐catalyzed properties of nanomaterials to design related nanomedicines. For example, Xu et al. designed and developed LaCoO₃ (LCO), a nanocrystal with multienzyme activity. LCO nanocrystals exhibit catalytic activities of peroxidase, oxidase, catalase, and glutathione peroxidase, which can generate a large amount of reactive oxygen species (ROS) in TME, reverse hypoxic microenvironments, and consume GSH, enhancing the sensitivity of tumor cells to ROS. The study further utilized ultrasound to accelerate the enzyme kinetic reaction and enhance ROS production. Meanwhile, La^3^⁺ ions released from LCO nanocrystals disrupted the lysosomal membrane, activated the caspase‐1/GSDMD pathway, and induced typical cellular pyroptosis. It was shown that LaCoO₃ nanocrystals could effectively inhibit tumor metastasis through its multienzyme catalytic activity, ultrasound‐enhanced effect, and induction of apoptosis (**Figure** **6**).^[^ ^70^ ^]^

The unique optical properties of nanomaterials enable their unique potential applications in phototherapy. Dong's team developed a multifunctional nanoplatform. The nanoplatform consists of gold NPs (Au NPs) core and a bilayer shell with an outer layer of metal organic framework (MOFs) and mesoporous silica. The study combined the photothermal therapeutic effect of nanomaterials by attaching ICGs on the surface of the NPs. The ICGs had excellent photothermal conversion properties. Under the irradiation of 808 nm near‐infrared (NIR) laser, ICG can rapidly convert light energy into heat energy to achieve local high temperature. The nanoplatform can directly damage the tumor cell membrane and internal structure through the generated high temperature, leading to the death of cancer cells. At the same time, the high temperature can also promote the release and penetration of drugs and enhance the efficacy of other drugs. Studies have shown that the nanoplatform combined with photothermal therapy (PTT) exhibits strong antitumor effects, with tumors significantly reduced in size or even partially disappearing.^[^ ^71^ ^]^

### 2.5 Diagnosis of Lung Cancer Metastasis

Rapid advances in nanomedicine have led to the development of new materials and methods for in vivo tracing and imaging diagnostics, offering the possibility of visual monitoring of lung cancer metastasis.^[^ ^72^, ^73^, ^74^, ^75^ ^]^ There are various types of nanoimaging materials currently under research for tumors, including metal nanomaterials, quantum dots, tungsten nanomaterials, carbon nanomaterials, and nanobodies. Nanoimaging technologies show higher sensitivity, better selectivity, and better real‐time monitoring, focusing on nanoimaging technology, nanosensor, nanofluorescent Probes, liquid biopsy technique, and cell separation technology.

For the early diagnosis of lymphatic metastasis of lung cancer, Chen et al. successfully developed a dual‐targeting nanoprobe, Au@Gd‐SiO2‐HA‐LyP‐1, which combines the photothermal properties of gold (Au), the stability of silicon dioxide (SiO2), the biocompatibility of HA, and the targeting ability of LyP‐1 peptide. In vitro cellular uptake experiments showed that the nanoprobe could be specifically taken up by lung cancer cells (A549) and lymphatic endothelial cells, while less uptake was observed for human umbilical vein endothelial cells and human peripheral blood mononuclear cells. In vitro T1‐weighted magnetic resonance imaging (MRI) experiments showed that Au@Gd‐SiO2‐HA‐LyP‐1 had stronger MRI signal enhancement compared to the commercial contrast agent Gd‐DTPA. In animal models, nanoprobes were found to accumulate predominantly in tumors and diseased lymphoid tissues and less in normal tissues by inductively coupled plasma mass spectrometry analysis. In vivo MRI imaging experiments showed that the MRI signals of tumors and diseased lymphoid tissues were significantly enhanced after injection of the nanoprobes, indicating that the nanoprobes have a good targeting imaging ability.Au@Gd‐SiO2‐HA‐LyP‐1 provides a new strategy for the early diagnosis and treatment of lymphatic metastasis in lung cancer, which has potential clinical applications.^[^ ^76^ ^]^

Moreover, nanotechnology has several other potential advantages, including good biosafety and biocompatibility,^[^ ^77^, ^78^, ^79^ ^]^ biodegradability,^[^ ^80^, ^81^, ^82^, ^83^, ^84^ ^]^ excellent serum stability,^[^ ^85^, ^86^, ^87^ ^]^ chemical inertness, improved solubility of poorly soluble drugs,^[^ ^88^ ^]^ altering the route of drug delivery, overcoming drug resistance,^[^ ^89^, ^90^, ^91^ ^]^ enhanced tissue penetration and reducing toxic side effects, which play an irreplaceable role in preventing lung cancer metastasis. The advantages of nanotechnology are summarized in **Table** **2**.

**Table 2:** List of advantages of nanotechnology inhibiting lung cancer metastasis.

Advantages	Specific classification	Representative nanomedicine
Multidrug codelivery	Codelivery of two drugs	LRT,^[^ ^92^ ^]^ Pt‐CUR @ PSPPN,^[^ ^93^ ^]^ Co‐NPs^[^ ^94^ ^]^
	Codelivery of three or more drugs	VF/S/A@CaP,^[^ ^33^ ^]^ AMMD^[^ ^71^ ^]^
	Carrier‐free nanodrugs	Cy‐TK‐LND NPs,^[^ ^95^ ^]^ MCO NVs,^[^ ^96^ ^]^ GC NPs^[^ ^97^ ^]^
Targeting	Passive targeting	PD‐NPs,^[^ ^98^ ^]^ PCPP,^[^ ^99^ ^]^ Cisplatin@GC‐p*K*s^[^ ^100^ ^]^
	Active targeting	ALN‐HA‐ZIF‐8@Sap,^[^ ^42^ ^]^ T12/P‐Lipo^[^ ^101^ ^]^
	Physicochemical targeting	Fe_3_O_4_‐Azo‐DOX Nanorobot‐ Collectives^[^ ^102^ ^]^
	Other targeting	AcPEI^[^ ^103^ ^]^
Controlled release	Responsive release to a single condition	SPN‐TAPP‐PCB4,^[^ ^104^ ^]^ APACP,^[^ ^105^ ^]^ DMNPN^[^ ^106^ ^]^
	Responsive release to multiple conditions	MOF@Ce6^[^ ^107^ ^]^
Synergistic antimetastasis effects	Photothermal therapy	LCO NPs^[^ ^108^ ^]^
	Photo dynamic therapy	Au@Gd‐SiO2‐HA‐Lyp‐1/DOX^[^ ^109^ ^]^
	Photoacoustic therapy	CEL@G‐SS‐NIR^[^ ^110^ ^]^
	Enzyme catalytic property	AP‐HAI^[^ ^111^ ^]^
Diagnosis of lung cancer metastasis	Nanosensor	T‐sense^[^ ^112^ ^]^
	Nanoimaging technology	CS‐ADH‐Rh‐LA^[^ ^113^ ^]^
	Nanofluorescent Probes	miR‐1^[^ ^114^ ^]^
	Liquid biopsy technique	EVs^[^ ^115^ ^]^
	Cell separation technology	On‐chip biomimetic model^[^ ^116^ ^]^
Biosafety and biocompatibility	–	UA/(AS‐IV)@PDA‐HA^[^ ^117^ ^]^
Biodegradability	–	TMCs^[^ ^118^ ^]^
Serum stability	–	CBSA /siRNA^[^ ^119^ ^]^
Chemical inertness	–	ERT@HMSNs/gel^[^ ^120^ ^]^
Solubility	–	p28‐NPs‐GEF^[^ ^121^ ^]^
Altering the route of drug delivery	–	CP@TDN^[^ ^122^ ^]^
Overcoming drug resistance	–	H‐MnO_2_ /DDP(Cy)‐TFA^[^ ^123^ ^]^
Enhanced tissue penetration	–	pDox^[^ ^124^ ^]^
Overcome toxic side effects	–	Lip‐CExo@PTX^[^ ^121^ ^]^

## 3 Promising NDDS for Prevention of Lung Cancer Metastasis

**Figure 7:**
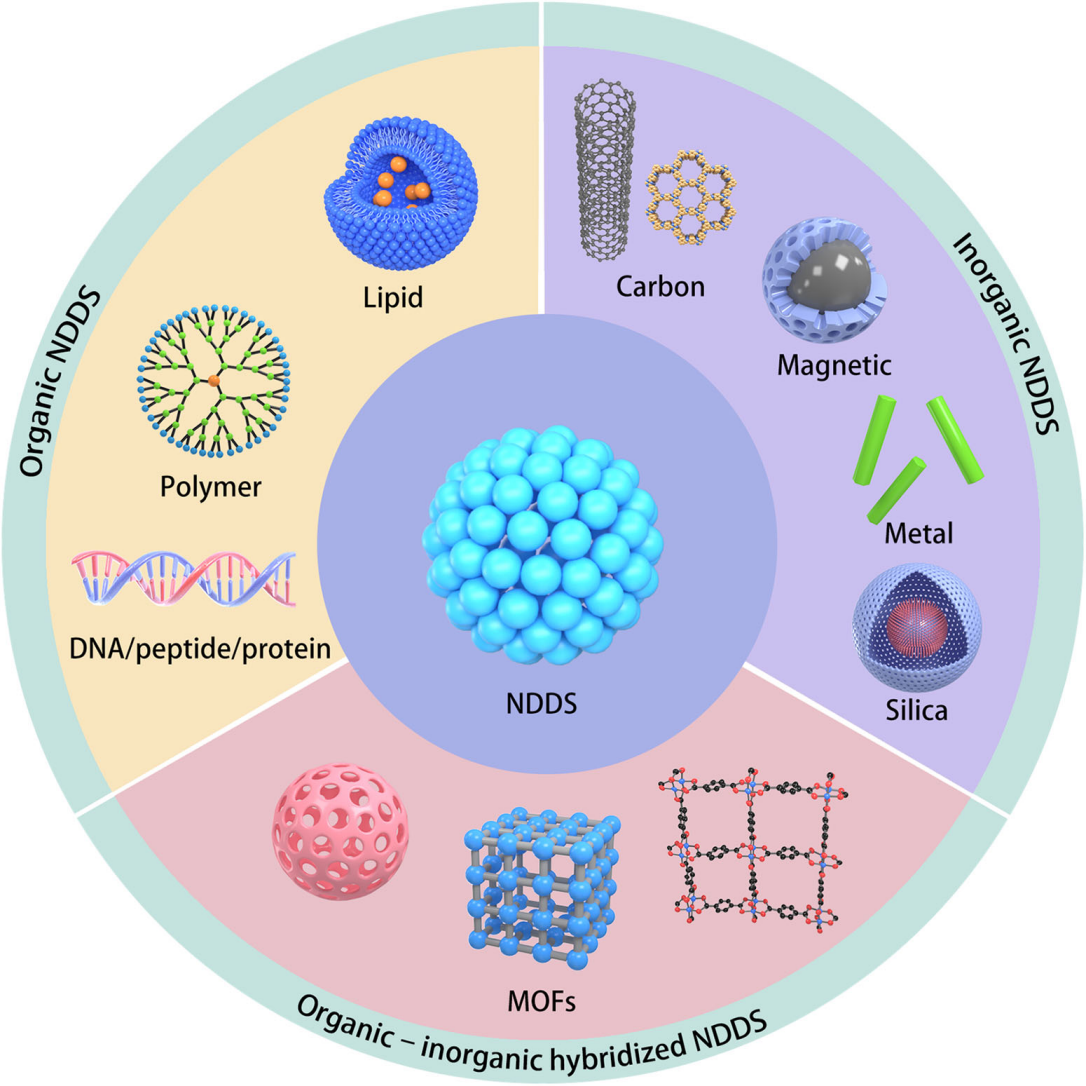
Nanodrug delivery systems (NDDS) for prevention of lung cancer metastasis based on the invasion‐metastasis cascade process.

Efficient and precise delivery of antilung cancer metastatic drugs can be achieved by designing and preparing suitable NDDS using nanomaterials. Currently, NDDS utilized for the prevention and treatment of lung cancer metastasis mainly include organic NDDS, inorganic NDDS, and organic–inorganic hybridized NDDS. Organic NDDS, including lipid‐based NDDS, polymer‐based NDDS, DNA/peptide/protein‐based NDDS, have been extensively investigated. Inorganic NDDS, including carbon‐based NDDS, magnetic‐based NDDS, metal‐based NDDS, and silica‐based NDDS, have been extensively investigated (**Figure** **7**). **Table** **3** details the characteristics and representatives of organic, inorganic, and organic–inorganic hybrid NDDS.

**Table 3:** Characteristics of promising nanodrug delivery systems (NDDS) for the prevention of lung cancer metastasis.

Types	Characteristics	Nanomaterials	Further types	Representatives
Organic	Advantages: good biocompatibility and degradability; precise targeting; adjustability of shape, size, surface properties, etc.; low synthesis cost. Disadvantages: limited drug‐carrying capacity; poor stability.	Lipid	Liposomes	T12/P‐Lipo,^[^ ^152^ ^]^ IND‐Lip^[^ ^153^ ^]^
			Solid lipid nanoparticles	SLN^[^ ^154^ ^]^
		Polymer	Polymer nanoparticles	SF NPs^[^ ^155^ ^]^
			Polymer nanomicelles	ZnPC‐M^[^ ^156^ ^]^
			Polymer nanofibers	poly(I:C)+R848‐prot‐NCs^[^ ^157^ ^]^
			Polymer nanocapsules	PMC^[^ ^158^ ^]^
		DNA/peptide/protein	DNA‐based NDDS	DAU@TDN‐p28‐s6‐siRNA^[^ ^159^ ^]^
			Peptide‐based NDDS	RGD@Cis⊂nZIF‐90^[^ ^160^ ^]^
			Protein‐based NDDS	CTX‐OLA‐ALB‐NPs^[^ ^161^ ^]^
Inorganic	Advantages: good stability; high drug‐carrying capacity; adjustability of shape, size, surface properties, etc.; good biocompatibility Disadvantages: poor biodegradability; poor targeting; high synthesis complexity; high synthesis cost; potential toxicity.	Carbon	Carbon nanotubes	CNT‐DOX^[^ ^162^ ^]^
			Graphene	Amine‐N‐GQDs^[^ ^163^ ^]^
			Nanodiamonds	FeND^[^ ^164^ ^]^
			Carbon dots	NCDs^[^ ^165^ ^]^
		Magnetic	Magnetic composites consisting of metals such as iron, nickel, cobalt, and their oxides	MNPs‐HA‐MTX,^[^ ^166^ ^]^ FRET^[^ ^167^ ^]^
		Metal	Gold, silver, and copper nanoparticles	HP‐Ag/Pt/NGR^[^ ^168^ ^]^
		Silica	Silica NDDS	DHUOCl‐25^[^ ^169^ ^]^
Organic–inorganic hybridized	Advantages: multifunctionality; enhanced drug loading and controlled release; improved biocompatibility and stability; enhanced targeting and therapeutic efficacy Disadvantages: complexity of preparation; higher cost; potential toxicity; difficulty of characterization and standardization.	–	Core–shell structures	MOF@HOF^[^ ^170^ ^]^
			Hybrid matrices	OIHMH^[^ ^171^ ^]^
			Surface modifications	Au‐RGD‐miR‐320a^[^ ^172^ ^]^
			Layered structures	MOF@MD‐D^[^ ^173^ ^]^
			Porous structures	HUIO‐66‐FDP^[^ ^174^ ^]^
			Dendritic macromolecular complexes	G5.NHAc‐Toy@TF^[^ ^175^ ^]^
			Nanogels	NDel 1^[^ ^176^ ^]^
			MOFs	Cis@MOF‐siVEGF^[^ ^177^ ^]^

### 3.1 Organic NDDS

#### 3.1.1 Lipid‐Based NDDS

Currently, lipid‐based NDDS focus on liposomes and solid lipid NPs. Liposomes are spherical, closed vesicles composed of phospholipid bilayer membranes that are similar to biological membranes. Liposomes can dissolve/carry hydrophobic, hydrophilic, and amphiphilic drugs which can be embedded in the inner core or lipid bilayer and then accumulate at the tumor site by passive targeting, active targeting, physicochemical targeting or other targeting, releasing the encapsulated drug into the cell by cytosolic drinking and endocytosis. To date, liposomes are widely used drug‐delivery vehicles in clinical practice, with numerous advantages, such as good biocompatibility, low toxicity, biodegradability, nonimmunogenicity, and easy surface functionalization. Several liposome formulations have been approved for marketing, including PTX, doxorubicin, and irinotecan liposomes, which are widely used in the clinical treatment of lung cancer and its metastasis.^[^ ^125^, ^126^ ^]^

However, liposomes are prone to rupture in the aqueous phase and exhibit fast drug release. A new generation of subparticles called solid lipid NPs was developed in the early 1990s to overcome the disadvantages of poor stability and fast drug release from liposomes.^[^ ^127^ ^]^ Solid lipid NPs are solid gel delivery systems made from naturally occurring or synthetic solid lipids as carriers, with the drug embedded in the lipid core or adhering to the lipid surface. Solid lipid NPs have higher stability, better controlled release properties, higher drug loading capacity, and lower toxicity. However, the preparation of solid liposomal NPs is more complicated than that of liposomes. In addition, compared with liposomes, solid liposome NPs have lower biocompatibility, limited targeting, lower drug encapsulation, and incomplete drug release.^[^ ^128^, ^129^ ^]^

#### 3.1.2 Polymer NDDS

Lipid‐based NDDS (such as liposomes and lipid NPs are the most common type of food and drug administration‐approved (FDA‐approved) NDDS because of their simple formulation, biocompatibility, and high bioavailability. Compared to liposomal nanomaterials, polymeric nanomaterials have been studied relatively late, only beginning to receive attention around the late 1980s. Polymer‐based NDDS are nanoscale polymers made from polymer materials through different preparation processes. Compared with lipid‐based NDDS, polymer‐based NDDS have advantages of include material diversity, high stability, excellent controlled release properties, and functionalization potential for complex drug delivery needs.^[^ ^130^, ^131^ ^]^ However, it may have disadvantages, such as toxicity, high complexity of preparation, higher cost, and incomplete drug release.

Polymer NPs, nanomicelles, nanofibers, and nanocapsules are the four most prominent polymer‐based NDDS. Polymer NPs are nanoscale particles composed of polymeric materials, and the commonly used polymers include polyvinyl alcohol,^[^ ^132^, ^133^ ^]^ polylactic acid,^[^ ^134^ ^]^ and poly (lactic‐*co*‐glycolic acid). These polymer NPs have promising biocompatibility and tunable physicochemical properties; thus, they can be used as carriers of biologically active substances, such as drugs and genes. Polymer nanomicelles are nanoscale micellar structures formed by the self‐assembly of water‐soluble polymers.^[^ ^135^ ^]^ Polymer fibers have good stability and drug encapsulation ability and can be used for the targeted delivery and controlled release of drugs.^[^ ^136^ ^]^ Polymer nanofibers are nanoscale fibrous structures prepared from polymeric materials. Owing to their high specific surface area and porous structure, they can be used for drug adsorption and controlled release and can be used in tissue engineering and as biosensors. Polymer nanocapsules are nanoscale capsule structures encapsulated in polymer materials that contain drugs, genes, and other bioactive substances. Polymer nanocapsules have good stability and controlled release properties and can be used for the targeted delivery and controlled release of drugs.^[^ ^137^ ^]^

#### 3.1.3 DNA/Peptide/Protein‐Based NDDS

NDDS based on DNA, peptides, and proteins are relatively innovative NDDS. As natural products, they are biologically active and therefore have better biocompatibility and low toxicity. In addition, such NDDS can be customized to suit different drug properties and therapeutic needs, which is highly flexible and innovative.

DNA molecules are 1D structures with high precision, highly controllable Watson–Crick base pairs, and delicate structures. DNA sequences can be precisely designed and regulated using synthetic techniques. By varying parameters, such as the size, shape, and surface modification of DNA nanostructures, they can be designed into complex nanodrug delivery lines with specific purposes to meet different delivery needs. Currently, they are primarily used in gene therapy. For example, a novel approach to DNA self‐assembly, DNA origami technology,^[^ ^138^ ^]^ has been proposed in recent years. Using the special structure of DNA molecules and the rules of base‐complementary pairing, specific regions of the long strands of natural DNA were folded and fixed with short strands to create specific structures with the desired function. Peptide‐based NDDS comprise peptide molecules that can be tuned to achieve different functions by adjusting the peptide sequence and assembly.^[^ ^139^, ^140^ ^]^ Protein‐based NDDS consist of protein molecules that form nanocarriers through self‐assembly or in combination with other materials.^[^ ^141^, ^142^, ^143^ ^]^ Their structures are complex and diverse, and can be structurally adapted to achieve different delivery effects.

Owing to the diversity of DNA/peptide/protein‐based nanostructures, good biofunctionality, ease of degradation, and nontoxicity of the degradation products, DNA/peptide/protein‐based nanomaterials have gradually become a research hotspot in the field of tumor prevention and treatment. To summarize, DNA, peptide and protein‐based NDDS, as an innovative NDDS, has some disadvantages despite its many advantages. For example, biomolecules, such as DNA, peptides, and proteins may trigger an immune response in the body, leading to inefficient drug delivery or adverse reactions. Their preparation usually involves complex chemical reactions and physical processes, requiring high‐precision equipment and strict process control. At present, such NDDS are still in the development stage and have not yet reached a fully mature state.

### 3.2 Inorganic NDDS

#### 3.2.1 Carbon‐Based NDDS

The preparation of fullerenes in 1985 opened up the field of carbon nanolera. Currently, the most widely researched carbon‐based NDDS include carbon nanotubes, graphene, nanodiamonds, and carbon dots. Carbon‐based NDDS have large specific surface areas and void structures for effective drug adsorption and storage. In addition, they have good biocompatibility and are resistant to degradation in the body allowing for sustained drug delivery over extended periods. Moreover, the carbon‐based NDDS could achieve PTT for lung cancer primary and metastatic foci through good PTT and photo dynamic therapy (PDT).^[^ ^144^ ^]^ However, unmodified carbon‐based materials may be recognized by the immune system, triggering an immune response, and metal catalysts or other impurities may remain during degradation. Therefore, they are potentially biotoxic and their biosafety still needs further research.

#### 3.2.2 Magnetic NDDS

##### Magnetic‐Based NDDS

Classical magnetic nanomaterials are magnetic composites consisting of metals, such as iron, nickel, cobalt, and their oxides. Magnetic nanomaterials are widely used in magnetically induced cancer therapies. Magnetic targeting technology can direct drugs to tumor sites for aggregation and responsive release with high efficiency and low toxicity.^[^ ^145^ ^]^ They also convert external magnetic fields into local stimulation signals, such as heat, electricity, and mechanical forces,^[^ ^146^ ^]^ which help the drugs kill tumor cells.^[^ ^147^ ^]^ Compared with other materials, magnetic nanomaterials are magnetically responsive, which makes them easy to separate and manipulate; they are small in size and have a large specific surface area, which is conducive to the increase of surface active sites and adsorption capacity; they are easy to be surface‐modified, and can be combined with other functional materials to form composites with multiple properties. However, magnetic nanomaterials are prone to agglomeration, which affects dispersion and stability; have fewer functional groups on the surface, which may affect their interactions with other molecules and their functionality; tend to lose their magnetic properties in acidic environments; are prone to oxidation, which leads to a decrease in adsorption capacity; and may produce a certain degree of toxicity in biological organisms, which requires rigorous toxicological assessment and safety verification.

##### Metal‐Based NDDS

Because metal NPs, such as gold, silver, and copper have good biocompatibility, they function as excellent drug carriers. Metallic NPs can be used to modulate the release rate and targeting of drugs by changing their surface properties and size to improve drug delivery efficiency. Metallic‐based NDDS can be used not only for drug delivery, but also in combination with imaging techniques, such as MRI, for integrated diagnosis and treatment of lung cancer. For example, gold is one of the most important metallic materials in nanostructural research, and its unique physical and chemical properties allow it to show extraordinary potential at the nanoscale. Gold‐based nanomaterials, covering a wide range of structures with different morphologies such as gold nanorods, gold nanostars, and gold nanocages. These materials not only inherit the excellent properties of gold itself, such as simple ductility and extremely high plasticity, but also show a series of brand‐new properties at the nanoscale. Among them, the optical properties of gold‐based nanomaterials are particularly outstanding. Due to the strong absorption and scattering of light by gold nanostructures, they exhibit significant surface plasmon resonance effects in the visible and NIR spectral range. This property makes gold‐based nanomaterials brilliant for applications in optical imaging, photothermal therapy, and photodynamic therapy in oncology. In addition, gold‐based nanomaterials have good catalytic activity, bioimaging, and other functions. However, despite the good biocompatibility of some of the metal‐based NDDS, there are still some safety risks. For example, prolonged retention in the body may trigger inflammatory reactions or other adverse effects. Currently, the clinical application of metal‐based NDDS in the prevention and treatment of lung cancer metastasis is still in the exploratory stage. More clinical studies and validation are needed to evaluate its efficacy and safety, as well as to determine the optimal treatment regimen and dosage.

##### Silica‐Based NDDS

Nanosilica is one of the most widely used nanomaterials worldwide. Silica nanomaterials have good biocompatibility and biodegradability and do not cause toxic side effects in the human body. Second, silica‐based NDDS have a large specific surface area, which can increase drug loading and release rates. In addition, the porous structure of the silica‐based NDDS can be controlled by adjusting the preparation conditions, which enables precise regulation of the drug release rate and delivery effect. With the continuous development and improvement of nanotechnology, silica‐based NDDS are expected to become important drug delivery platforms, providing more effective and safer drug delivery solutions for clinical treatments. Currently, Silica‐based NDDS have been extensively investigated.^[^ ^148^, ^149^, ^150^ ^]^ Although there are relatively few large‐scale clinical trials of silica‐based NDDS, some studies have entered the clinical trial stage, showing their potential for clinical applications.

### 3.3 Organic–Inorganic Hybridized NDDS

Organic nanomaterials exhibit good biosafety, biodegradability, and modifiability, whereas inorganic nanomaterials exhibit unique physicochemical properties and functional and structural diversity. Therefore, organic–inorganic hybrid nanomaterials developed by integrating these two materials combine the advantages of organic polymer materials and inorganic NPs and have broader application prospects. Organic–inorganic hybridized NDDS includes core–shell structures, hybrid matrices, surface modifications, layered structures, porous structures, dendritic, macromolecular complexes, nanogels, and MOFs. for example, Yaghi et al. successfully synthesized a compound with a MOF structure for the first time, marking the formal introduction of MOFs. Recently, MOFs have become a popular research topic in the fields of materials science and chemistry. MOFs are crystalline materials consisting of metal ions or metal clusters with organic ligands, highly ordered pore structures, and large specific surface areas that can be loaded with large amounts of drugs. In addition, they also have the characteristics of inorganic materials, such as multifunctionality, a highly controllable structure, good thermal and chemical stability, and organic nanomaterials with good biosafety and degradability. Therefore, they have a wide range of prospective applications in gas adsorption, separation, storage, and catalysis.^[^ ^151^ ^]^

## 4 Emerging Nanostrategies for Lung Cancer Metastasis Based on the Invasion‐Metastasis Cascade Process

Metastatic dissemination of aggressive lung cancer cells from the primary tumor to distant organs is a multifactorial, multistep, dynamic, and ordered complex pathological process called the invasion‐metastasis cascade. This complex biochemical and biological alteration is mostly dependent on cancer cells and their associated ECM. The entire process of the invasive‐metastatic cascade can be summarized in seven separate steps: 1) proliferation, local migration, and invasion of lung cancer cells,^[^ ^178^, ^179^ ^]^ 2) intravasation into the blood circulatory system,^[^ ^9^, ^180^ ^]^ 3) survival in the blood circulatory system,^[^ ^181^, ^182^ ^]^ 4) retention in the microvasculature of various organs; 5) extravasation of lung cancer cells and colonization in distant organ sites,^[^ ^183^, ^184^, ^185^ ^]^ 6) formation of micrometastasis; and 7) adaptation to the microenvironment of distant tissues and formation of large metastasis. Therefore, suppressing lung cancer metastasis is more complicated than treating primary lung cancer. Blocking the invasion‐metastasis cascade of lung cancer is key to improving the antimetastatic efficacy in patients. The advantages of nanotechnology and the functional diversification of nanomaterials described above provide new strategies and directions for the development of novel antimetastatic drugs against lung cancer. Designing individualized and precise nanomedicines for the invasive metastatic cascade of lung cancer can overcome the bottleneck of existing antimetastatic drugs and substantially improve the antimetastatic efficacy in lung cancer patients by accurately and effectively combating one or more metastatic mechanisms. Based on the invasion‐metastasis cascade process in lung cancer, this review discusses the emerging nanostrategies designed in the last several years against lung cancer metastasis (Figure 1). For better understanding, the antimetastasis nanostrategies were further categorized into the following three stages: 1) targeting premetastatic stages (steps 1–2); 2) targeting metastasizing stages (steps 3–4); and 3) targeting metastasized and colonization stages (steps 5–7).

### 4.1 Targeting Premetastatic Stages

Cancer cell proliferation, migration, invasion, and intravasation are the first stages of the invasion‐metastasis cascade in lung cancer. Invasive cancer cells are highly proliferative, break through the surrounding basement membrane, cross the ECM and stromal cells, penetrate the walls of blood and lymphatic vessels, and intravasate into the blood. Designing appropriate nanomedicines or using NDDS to efficiently deliver antimetastatic drugs targeting specific mechanisms in this phase can effectively arrest lung cancer metastasis. Herein, the two main nanostrategies, targeting invasive lung cancer cells and targeting the TME in primary foci, have been elaborated.^[^ ^186^ ^]^

#### 4.1.1 Targeting Invasive Cancer Cells

**Figure 8:**
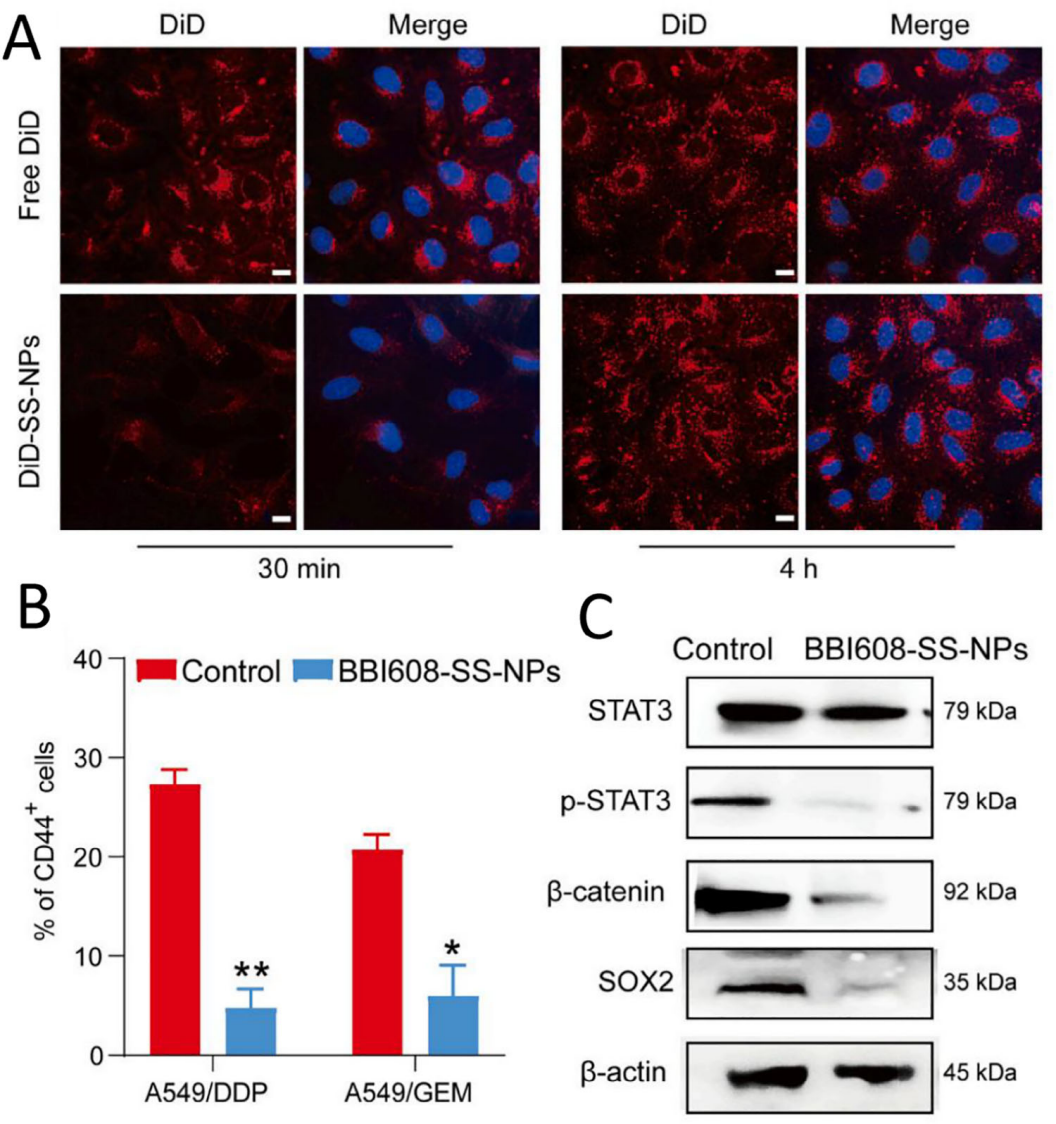
Nanostrategies inhibit lung cancer metastasis based on targeting invasive cancer cells. A) Representative confocal microscopic images showing cellular uptake of DiD‐labeled NPs (DiD‐SS‐NPs, red) in H1975 cells after 30‐min or 4‐h incubation. The nuclei were stained with DAPI (blue). Scale bar: 10 µm. The cellular uptake of DiD‐labeled NPs in both CSCs (CD44+) and non‐CSCs (CD44) after 30‐min or 4 h; B) Changes in the percentage of CD44+ cells in A549/DDP and A549/GEM cells treated with BBI608‐SS‐NPs (0.2 µm) for 48 h via flow cytometric analysis. C) Representative western blots for the expression of stemness‐related proteins in A549/DDP cells treated with BBI608‐SS‐NPs (0.2 µm). Data are expressed as the means ± SEM (*n* = 3), **p < 0.01, ***p < 0.001. Reproduced with permission.^[^ ^195^ ^]^ Copyright 2023, Elsevier.

Initiation of lung cancer metastasis requires an increase in the proliferative, migratory, and invasive capabilities of cells. Therefore, inhibiting invasive cancer cells is key to suppressing lung cancer metastasis. Lung cancer stem cells (LCSCs) are representative invasive cancer cells. Although LCSCs are only a very small subpopulation of tumor tissue, accounting for only ≈0.01%–2%, they have a high capacity for self‐renewal and multilineage differentiation.^[^ ^187^, ^188^, ^189^ ^]^ Total elimination of LCSCs or induction of the terminal differentiation of LCSCs into nonstem cells that are sensitive to conventional radiotherapy are potentially effective strategies. However, LCSCs are usually in a relatively quiescent cell cycle, and it is difficult for chemotherapeutic agents to exert cytotoxic effects on LCSCs in clinical settings. Researchers are committed to developing drugs with anti‐LCSCs activity.^[^ ^190^ ^]^ Unfortunately, however, to date, effective drugs targeting LCSCs have not been successfully translated into the clinic. BBI608, a small molecule inhibitor of the STAT3 signaling pathway, has been shown to be effective in inhibiting stemness gene expression in LCSCs, inducing cancer cell death, and suppressing cancer recurrence and metastasis. The researchers designed a redox‐responsive PEGylated branched polymer NPs (BBI608‐SS‐NPs) to deliver BBI608 in response to poor water solubility and nonspecific biodistribution of BBI608. They synthesized PEGylated branched polymers using *N*‐(2‐hydroxypropyl)methacrylamide and deoxycholic acid, and by evaporation, BBI608 was encapsulated into the polymer to form self‐assembled NPs. The results showed that BBI608‐SS‐NPs more potently inhibited LCSCs proliferation through improved drug solubility and tumor targeting compared to free BBI608. This study provides new ideas and methods for the development of drugs targeting LCSCs, and is expected to bring new breakthroughs for the prevention of lung cancer metastasis (**Figure** **8**).^[^ ^191^ ^]^ Several natural active ingredients that target LCSCs and exhibit anti‐LCSCs activity. However, these natural active ingredients often suffer from poor pharmacokinetic properties, such as low solubility, poor stability, low bioavailability, and difficulty in precise target delivery, all of which severely limit their widespread clinical use. The emergence of nanotechnology provides an effective means to solve this problem. Through nanotechnology, it is possible to encapsulate these natural active ingredients in nanocarriers, such as NPs, liposomes, polymer micelles, etc., thereby significantly improving their pharmacokinetic properties.^[^ ^192^ ^]^

Solid tumors have dense tumor tissue, high interstitial pressure, and heterogeneous vascular distribution, whereas LCSCs are often located deep in the tumor tissue. Therefore, it is difficult for drugs to penetrate deep into the tumor and specifically kill LCSC. Targeted and permeable drug delivery using NDDS can achieve deep tumor delivery and improve drug efficacy.^[^ ^193^ ^]^ Researchers prepared H‐MnO2 NPs and successfully loaded them with the NIR fluorescent dye ICG and the LCSCs‐targeting ingredient liquorice chalcone. The NPs have the advantages of pH‐responsiveness, high penetration ability and targeting, and can deeply penetrate into the interior of the tumor and significantly inhibit the proliferation and differentiation of LCSCs. Specifically, the small size and surface charge characteristics of the NPs help them penetrate into the tumor tissue and improve the bioavailability and therapeutic effect of the drug. Further, by modifying HA, the NPs were able to specifically target LCSCs with high expression of CD44 receptor and improve the deep penetration of the drug. Then, the H‐MnO2 NPs were able to responsively degrade in the acidic TME, releasing the loaded drug for targeted drug delivery. The results showed that this deep drug delivery strategy achieved efficient targeted therapy for LCSCs.^[^ ^194^ ^]^

LCSCs contribute to the heterogeneity of tumors owing to their ability to differentiate into heterogeneous cancer cells, resulting in the poor efficacy of single‐agent therapy.^[^ ^195^ ^]^ Therefore, combining multiple drugs with different anticancer mechanisms is an effective means of improving the efficacy of antilung cancer metastasis. NDDS can achieve multidrug codelivery to achieve dual or multiple killing effects on LCSCs. In addition, nanomaterials have unique chemical, mechanical, electrical, optical, magnetic, magneto‐optical, and electro‐optical properties that can be used for PTT, magneto‐thermal therapy, and radiotherapy under responsive stimulation. Therefore, nanomaterials may play a synergistic therapeutic role.

To perform precision strikes against LCSCs, noninvasive in vivo imaging techniques are needed to identify and evaluate LCSCs. However, LCSCs account for very little in tumors, making them difficult to detect. A nanoprobe is a probe prepared using nanotechnology, usually in the nanoscale. Such probes can be piggybacked with specific molecules or drugs to enable detection of individual living cells through precise targeting. It has a wide range of applications in cell biology and medicine. Dharmalingam et al. develop a multimodal nanoplatform (SERS) for the detection of epigenetic markers in tumor‐initiating cancer stem cells (tiCSCs). They used ultrafast laser ionization to introduce phosphorus dopant molecules into silicon NPs, which enhanced Raman scattering and enabled label‐free analysis of biomarkers in tiCSCs at cellular level resolution. The results showed that SERS successfully achieved the detection of epigenetic markers in tiCSCs and revealed their transformation signals after drug treatment.^[^ ^196^ ^]^

#### 4.1.2 Remodeling the TME in the Primary Foci

The TME is the internal environment in which the tumor cells exist and interact with surrounding noncancerous cells. TME is a complex ecosystem that includes tumor cells, immune cells, cancer‐associated fibroblasts (CAF),^[^ ^197^, ^198^, ^199^ ^]^ endothelial cells, mural cells, additional tissue‐resident cells, and the dynamic, vascularized ECM in which these cells are embedded. During tumor development, the TME is not a “silent bystander” but an “active promoter” of cancer progression. Therefore, therapeutic strategies for TME have gradually become a research hotspot in the field of tumor therapy. Altering the microenvironment of TME by regulating the cellular and noncellular components in TME can be expected to improve the efficacy of tumor therapy and bring new therapeutic hope to tumor patients. With the rapid development of nanotechnology, it plays a crucial role in assisting the remodeling of TME. With its unique size, surface and quantum effects, nanotechnology has revolutionized the fields of drug delivery, cellular imaging, and biosensing. In tumor therapy, nanotechnology can be used to design intelligent drug delivery systems that can precisely identify and target specific cells or molecules in TME to achieve targeted release of drugs, thus maximizing the therapeutic effect of the drugs while minimizing damage to normal tissues.

##### Inhibiting the Generation of Tumor Neovascularization Vessels

TME includes rich neovascularizations,^[^ ^200^ ^]^ which are incomplete and leaky, making them more susceptible to allowing tumor cells to enter the blood than mature vessels. Inhibition of tumor neovascularization is an effective method to achieve metastatic suppression in lung cancer.^[^ ^201^ ^]^ Antiangiogenic agents widely used to treat lung cancer include vascular endothelial growth factor (VEGF) inhibitors (bevacizumab), recombinant human vascular endothelial inhibitors, and small‐molecule multitargeted tyrosine kinase inhibitors (amilorotinib hydrochloride). These antiangiogenic drugs are designed to target existing tumor‐infiltrating blood vessels and inhibit the formation of newly formed blood vessels, thereby interrupting tumor metabolism and growth. When used alone or in combination with chemotherapy and radiotherapy, it has efficient clinical efficacy and can prolong the survival of lung cancer patients. However, addressing the adverse effects of ant vascular drugs, such as toxicity (hypertension, cardiotoxicity, and proteinuria), drug resistance, and the upregulation of various proangiogenic signals, remain challenging. which substantially limits their clinical efficacy. Rapid advances in nanotechnology offer the potential to address the limitations of current antiangiogenic treatments.^[^ ^202^ ^]^

**Figure 9:**
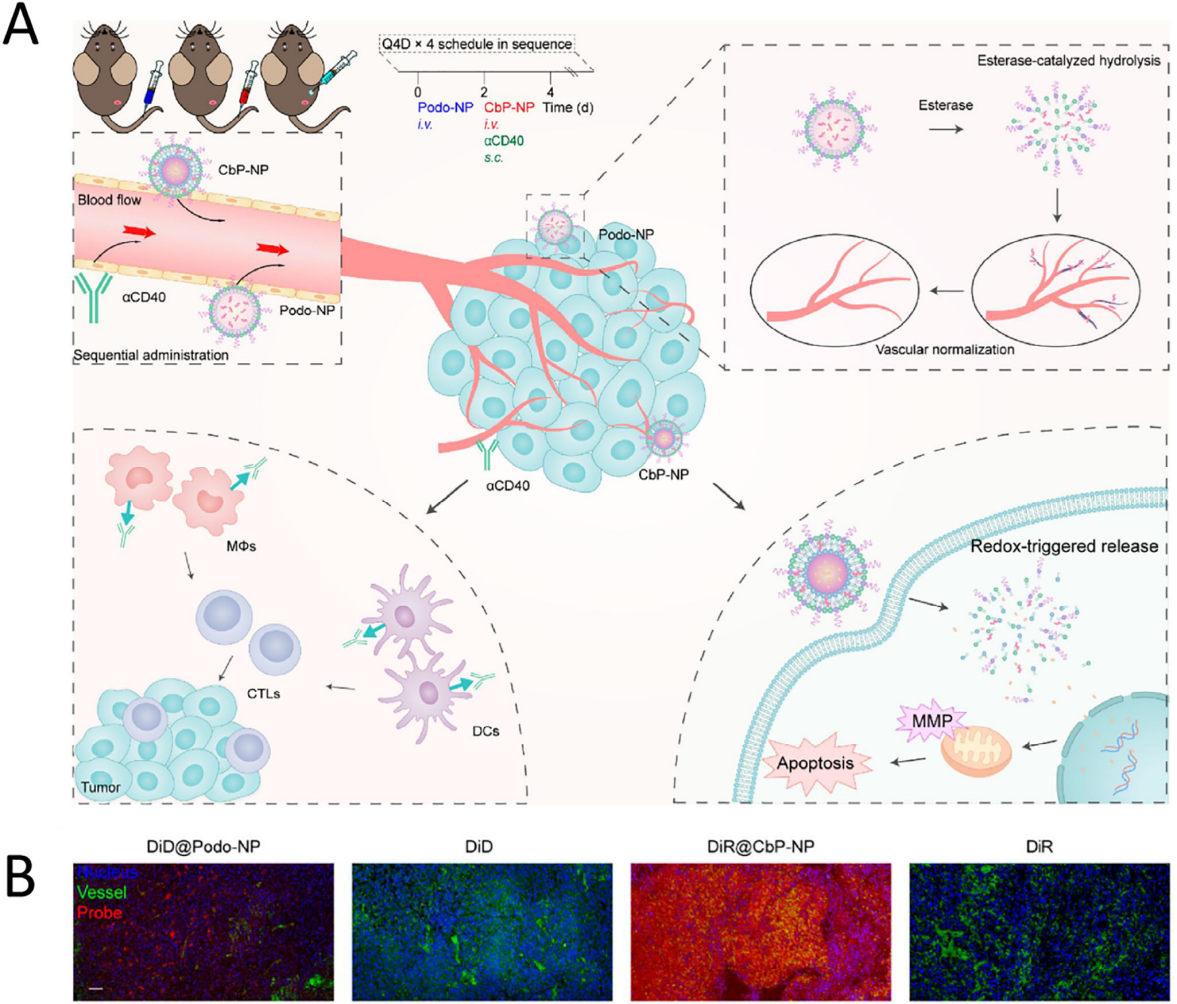
Nanostrategies inhibit lung cancer metastasis through inhibiting the generation of tumor neovascularization vessels. A) Schematic structure and mechanism of nanomedicine. B) The average number of microvessel outgrowths of aortic ring fragments treated with free drugs or nanoparticles. C) The effect of free drugs or NPs on the interconnected network. D) The blood vessel length of CAMs treated with free drugs or NPs. The equivalent Podo and Pt concentrations were 2 and 20 µm, respectively. E) Colocalization of DiD@Podo‐NP, DiD, DiR@CbP‐NP, or DiR in tumors with nuclei stained with DAPI and microvessels stained with antibody against CD31 (scale bar = 50 µm). Reproduced with permission.^[^ ^208^ ^]^ Copyright 2021, American Chemical Society.

Itraconazole (ITA), an antifungal drug with potent antiangiogenic activity, was coencapsulated with PTX in PEG‐PLA micelles by Zhang et al. The coencapsulated micelles showed potent antiangiogenic and antiproliferative abilities against NSCLC cells in vitro and significantly improved intra‐tumor drug accumulation in vivo. Compared with PTX monotherapy or combination therapy using PTX and ITA micelles alone, co‐encapsulated micelles showed strikingly superior efficacy in inhibiting tumor growth and preventing recurrence and metastasis in Kras mutation patient‐derived xenografts, orthotopic xenograft models, and PTX sensitivity.^[^ ^203^ ^]^ Ling et al. constructed a species of bioresponsive NPs containing podophylotoxin (Podo), the Podo‐NPs. Podo‐NPs inhibited angiogenesis by blocking endothelial cell proliferation and migration, neointimal sprouting, tubule formation, and neointimal stabilization. VEGF blockade and endothelin stimulation normalize the tortuous tumor vascular system to allow effective infiltration of effector immune cells, regression of transplanted tumors, and inhibition of disseminated tumors in a mouse model of lung cancer (**Figure** **9**).^[^ ^204^ ^]^

##### Remodeling the Immune‐Microenvironment

**Figure 10:**
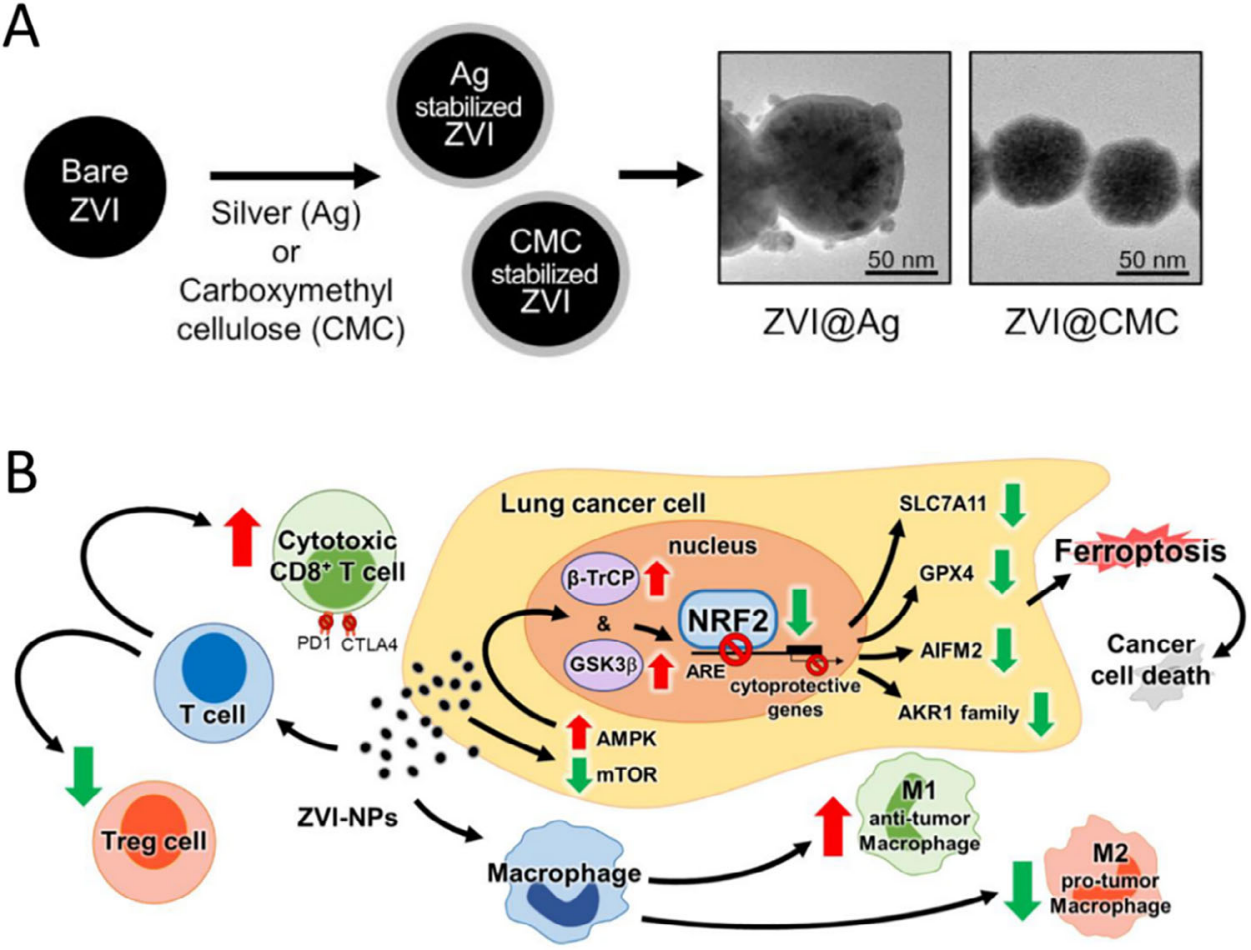
Nanostrategies inhibit lung cancer metastasis through immune‐microenvironment remodeling. A) Transmission electron microscopy images showing the round shape morphology and surface coating of both types of nanoparticles. Scale bar: 50 nm. B) Model of the dual synergistic anticancer activities of ZVI‐NPs. Reproduced with permission.^[^ ^209^ ^]^ Copyright 2021, Ivyspring International.

Relevant studies have shown that TME presents an immunosuppressive state. Tumor cells inhibit the activity and function of immune cells through various mechanisms, including the secretion of immunosuppressive factors (TGF‐β, IL‐10, etc.) and the up‐regulation of the expression of immune checkpoint molecules (PD‐1, CTLA‐4, etc.). These mechanisms work together to render TME immunosuppressive, which is conducive to tumor cell growth and metastasis. To address the immunosuppressive TME, scientists are exploring a variety of therapeutic strategies, and immune checkpoint inhibitors, immune cell therapy, etc., have achieved greater clinical benefit. However, immunotherapy for lung cancer has many problems, such as immune‐related adverse reactions and drug resistance, which seriously affect its clinical efficacy. Hsieh et al. developed zero‐valent iron NPs (ZVI‐NP), including silver‐coated (ZVI@Ag) and carboxymethylcellulose‐coated (ZVI@CMC) types. ZVI‐NP exerts its anticancer effects through a dual mechanism: first, it is by inducing iron death in cancer cells:ZVI‐NP induces iron death in lung cancer cells by inducing mitochondrial dysfunction, intracellular ZVI‐NP induces iron death in lung cancer cells by inducing mitochondrial dysfunction, intracellular oxidative stress, and lipid peroxidation: ZVI‐NP triggers iron death by activating GSK3β/β‐TrCP‐dependent NRF2 degradation through the AMPK/mTOR signaling pathway. Second, it enhances antitumor immune response by regulating the tumor immune microenvironment: ZVI‐NP transforms tumor‐promoting M2 macrophages into antitumor M1 macrophages, reduces the proportion of regulatory T cells (Tregs), and down‐regulates the expression of PD‐1 and CTLA4 in CD8^+^ T cells, which enhances their cytotoxic activity, while attenuating the expression of PD‐L1 in cancer cells. The study confirmed that ZVI‐NP significantly inhibited lung cancer metastasis in vivo (**Figure** **10**).^[^ ^205^ ^]^

**Figure 11:**
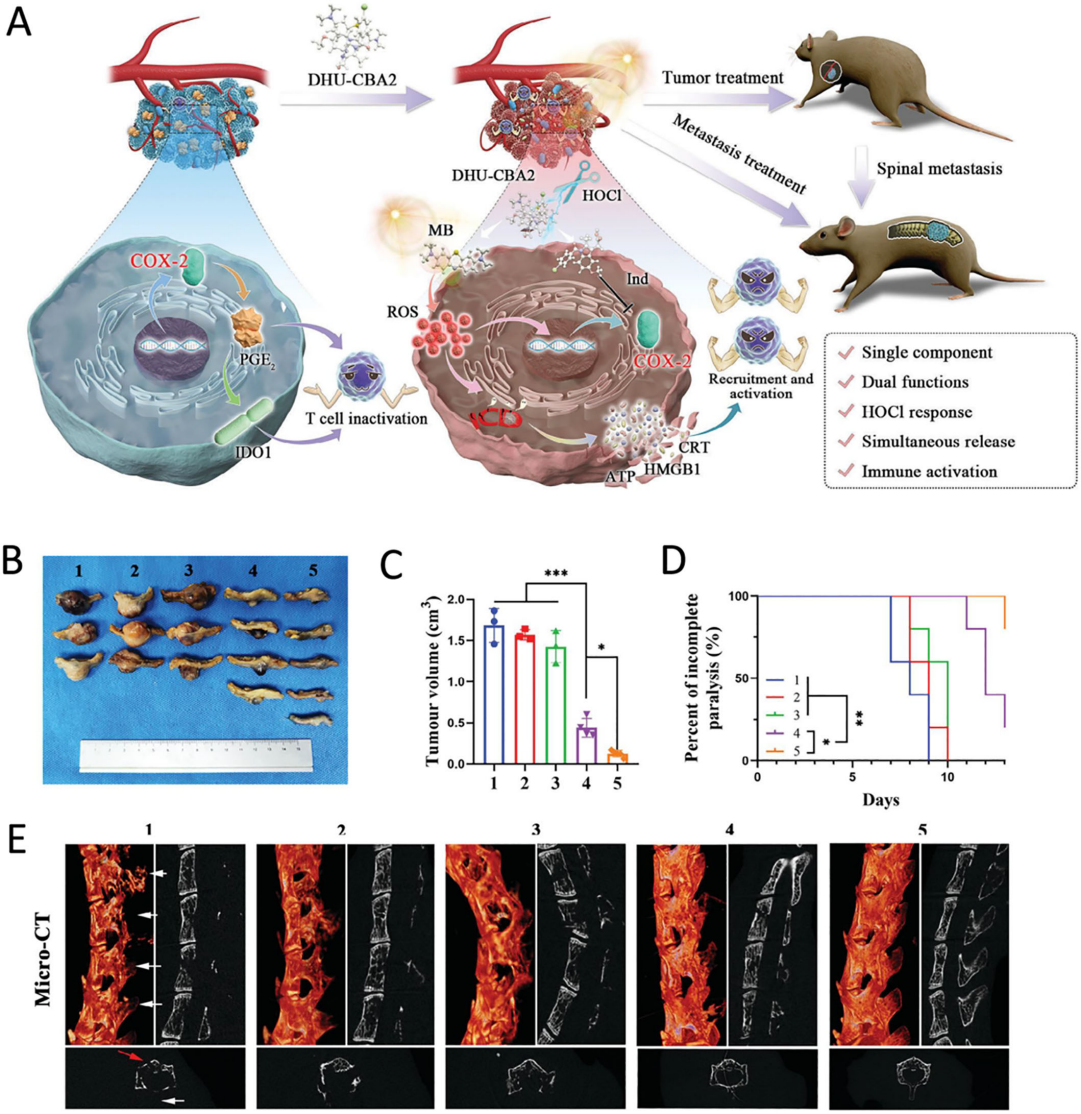
Nanostrategies inhibit lung cancer metastasis through immune‐microenvironment remodeling. A) Schematic illustration of the concept and effective mechanism by which DHU‐CBA2 triggers immunogenic cell death and blocks immune escape to remodel the tumor microenvironment. B) Photographs of the tumors and C) tumor volume at the end of the observation period after various treatments (1: CTRL; 2: DHUCBA3; 3: DHU‐CBA2; 4: DHU‐CBA3 (+); 5: DHU‐CBA2 (+); “(+)” represents laser irradiation). Experimental data in (B) are presented as the mean ± SD. Statistical significance was calculated via one‐way analysis of variance with Tukey's test (*n* = 3–5 individual animals per group, *p < 0.05, **p < 0.01, ***p < 0.001). D) Paralysis rate monitoring. Statistical significance was calculated via a log‐rank test for comparison (*n* = 5 individual animals per group, *p < 0.05, **p < 0.01, ***p < 0.001). E) 3D and planar view reconstruction images of spines showing the osteolytic vertebral plate (white arrow) and anterior centrum (red arrow) at the end of the observation period. Reproduced with permission.^[^ ^210^ ^]^ Copyright 2024, Wiley.

Wang et al. develop a single‐component bifunctional self‐enhancement strategy. Using methylene blue as a photosensitizer and indomethacin as a cyclooxygenase‐2 inhibitor linked by a hydrazide bond, they synthesized a prodrug, DHU‐CBA2, that responds to hypochlorous acid generated by the TME. DHU‐CBA2, by combining PDT and cyclooxygenase‐2 inhibition, can improve the immunosuppressed TME and enhance the therapeutic efficacy of lung cancer and its spinal metastasis (**Figure** **11**).^[^ ^206^ ^]^

##### Regulating Metabolic Reprogramming

Metabolic reprogramming is present in TME. In normal lung cells, metabolic pathways are tightly regulated to ensure that the cells maintain basic life activities while also responding adaptively to changes in the internal and external environment. However, in lung cancer cells, this metabolic regulatory mechanism is significantly altered, leading to metabolic reprogramming. Metabolic reprogramming provides lung cancer cells with large amounts of energy and substances. Lung cancer cells preferentially produce energy through glycolysis by enhancing the glycolytic pathway, even under aerobic conditions, a process known as the “Warburg effect.” This metabolic approach allows lung cancer cells to rapidly convert glucose to lactate and in the process produce large amounts of ATP (adenosine triphosphate), which provides energy for rapid cell proliferation. In addition to energy supply, metabolic reprogramming also provides lung cancer cells with substances needed for the synthesis of biomolecules. For example, by enhancing the pentose phosphate pathway, lung cancer cells are able to produce large amounts of nucleotides and reduced coenzyme II (NADPH), which are essential for the synthesis of DNA, RNA, and lipids. Also, by upregulating the fatty acid synthesis pathway, lung cancer cells increase the synthesis of fatty acids, which provide the necessary lipid components for cell membrane construction and signaling.

**Figure 12:**
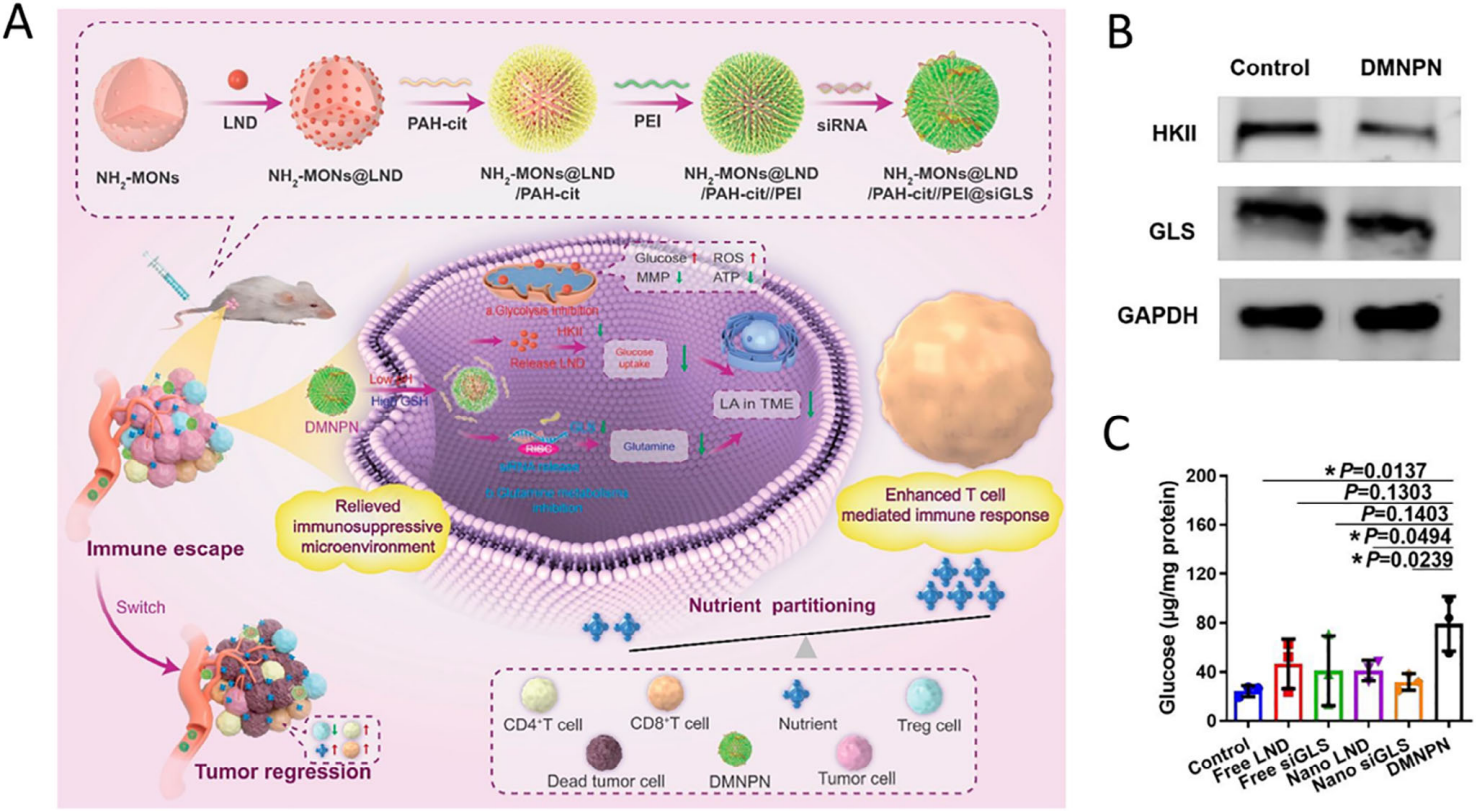
Nanostrategies inhibit lung cancer metastasis through regulating metabolic reprogramming. A) Schematic of a dual‐mechanism based nutrient reallocation strategy for enhanced immunotherapy against anti‐PD‐1 resistant tumors. B) Representative western blot images of protein expression in 4T1 cells after DMNPN treatment for 24 h. C) Glucose levels in 4T1 cells treated with different formulations for 24 h. The data are presented as the mean ± SD (*n* = 3). *p < 0.05, **p < 0.01, ***p < 0.001, and ****p < 0.0001. Reproduced with permission.^[^ ^212^ ^]^ Copyright 2023, American Chemical Society.

Yu et al. constructed a cascade of enzyme‐driven nanomotors (NM‐si) that could simultaneously provide sufficient oxygen to deep tumors and inhibit aerobic glycolysis to enhance antimetastasis in chemotherapy. Catalase and glucose oxidase were coadsorbed onto gold CAuNCs@HA to form an autodriven nanomotor (NM), and hexokinase‐2 small interfering RNA (siRNA) is incorporated into the nanomotor as (NM‐si)_2._ The oxygen gradient created by the nanomotor helps alleviate hypoxia in deep tumors. Moreover, autoreactivity promotes NM‐si and lysosomal escape and effectively knocks down HK‐2 to inhibit glycolysis. The in vivo results showed promising antimetastatic effects of commercially available albumin‐conjugated PTX after pretreatment with NM‐si for TME reconstruction. This cascade of enzyme‐driven nanomotors offers potential promise in reversing hypoxic TME and metabolic pathways to enhance antimetastasis of chemotherapy.^[^ ^207^ ^]^ Competitive nutrient depletion between rapidly proliferating cancer cells and T cells leads to nutrient deprivation of the immunosuppressive TME and T cells, resulting in low response rates and resistance to immunotherapy. Zhang et al. proposed a DMNPN that can simultaneously modulate the immunosuppressive TME, which consists of charge‐reversal biodegradable mesoporous silica, the encapsulated glycolysis inhibitor lornidamine, and siRNA against glutaminase. By inhibiting glycolysis to reduce lactate production and downregulating glutaminase expression to reduce glutamine uptake by the tumor cells, DMNPN can effectively remodel metabolism and nutrient partitioning to attenuate the immunosuppressive TME and increase the nutrient availability for T cells, thereby enhancing antitumor immunity. This nutrient partitioning nanomodulator can effectively inhibit the growth of anti‐PD‐1‐resistant tumors and prevent metastasis and recurrence (**Figure** **12**).^[^ ^106^ ^]^

##### Targeting ECM and Stromal Cells

**Figure 13:**
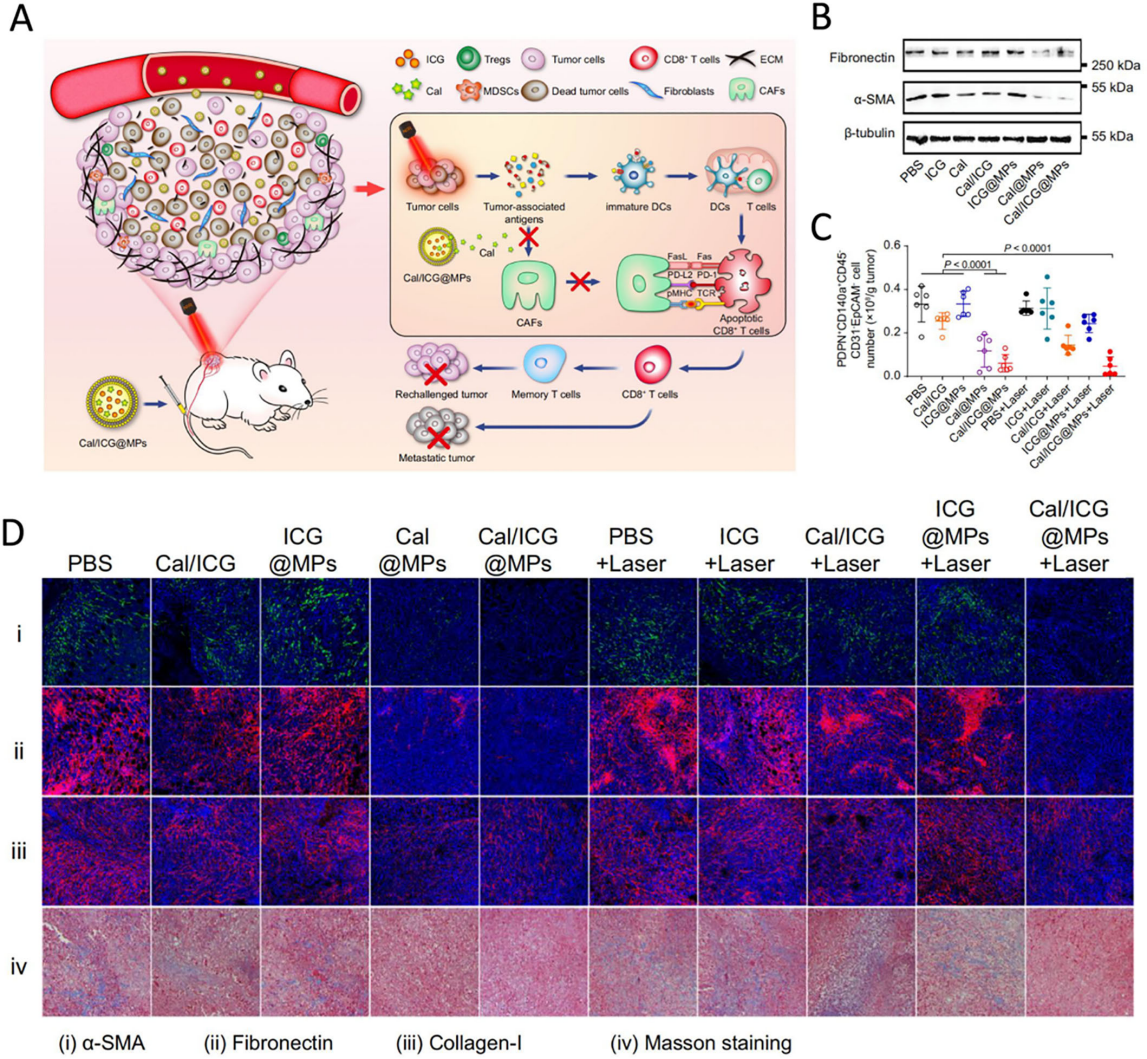
Nanostrategies inhibit lung cancer metastasis based on targeting extracellular matrix and stromal cells. A) Scheme of Cal/ICG@MPs as an efficient drug to regulate CAFs to enhance the efficacy of photothermal therapy. B) Western blot analysis of fibronectin and α‐SMA expression in myofibroblasts after treatment with PBS, ICG, Cal, Cal/ICG, ICG@MPs, Cal@MPs, or Cal/ICG@MPs derived from tumor cells at the ICG concentration of 4 µg mL^−1^ and Cal concentration of 60 ng mL^−1^ for 48 h. The images are representative of three independent samples. C) Numbers of CAFs in tumor tissues of stroma‐rich tumor‐bearing mice at 14 days after intravenous injection of PBS, ICG, Cal/ICG, ICG@MPs, Cal@MPs, or Cal/ICG@MPs at the ICG dosage of 8 mg kg^−1^ and Cal dosage of 120 µg kg^−1^ twice every 2 days, followed with or without 808 nm laser irradiation (1.5 W cm^−2^, 10 min) at 2 h after the last injection. Data are presented as the means ± SD (*n* = 6 mice per group; one‐way analysis of variance followed by Tukey's honestly significant difference post hoc test). D) Immunofluorescence staining of α‐SMA, fibronectin, and collagen‐I, and Masson's trichrome staining of collagen in tumor sections of stroma‐rich tumor‐bearing mice after treatment indicated in (C). The images are representative of three biologically independent mice. Reproduced with permission.^[^ ^213^ ^]^ Copyright 2022, Nature Publishing Group.

ECM and stromal cells play a crucial role in lung cancer metastasis, and they are key factors influencing antimetastatic efficacy. ECM and stromal cells, such as CAFs, and tumor‐associated macrophages promote lung cancer metastasis by providing a physical barrier, modulating cellular behavior, promoting tumor angiogenesis, and influencing drug distribution. For example, Yang's team focused on breaking the physical barrier effect of CAFs, enhancing the accumulation and penetration of photosensitizers in tumor tissues, and achieving efficient PTT and PDT. The team developed a tumor cell‐derived particles (MPs) coloaded with calcipotriol and ICG (called Cal/ICG@MPs). Experiments validated the modulatory effects of Cal/ICG@MPs on CAFs, including reduction of tumor ECM, enhancement of ICG accumulation, and penetration in tumors, induction of immunogenic cell death in tumor cells, promotion of dendritic cell maturation and CD8^+^ T cell activation (**Figure** **13**).^[^ ^208^ ^]^

### 4.2 Targeting Metastasizing Stages

Invasive cancer cells from the primary foci intravasate into the proximal vasculature or lymphatic system and reach the target organs via the peripheral circulation. These cells are collectively referred to as circulating tumor cells. This is the second stage of the invasion‐metastasis cascade of lung cancer. During circulation, circulating tumor cells (CTCs) undergo apoptosis or are phagocytosed due to physical, redox, and immune surveillance, with only a small number of surviving CTCs. These surviving CTCs circulate as individual cells or microclusters enriched with stem cell‐like cancer cells and colonize suitable premetastatic ecological sites. At this stage, effective killing of CTCs becomes a central strategy. Currently, nanostrategies for this critical link focus on two main aspects: 1) Isolation and detection of CTCs; 2) Targeting and killing of CTCs.

#### 4.2.1 Isolation and Detection of CTCs

**Figure 14:**
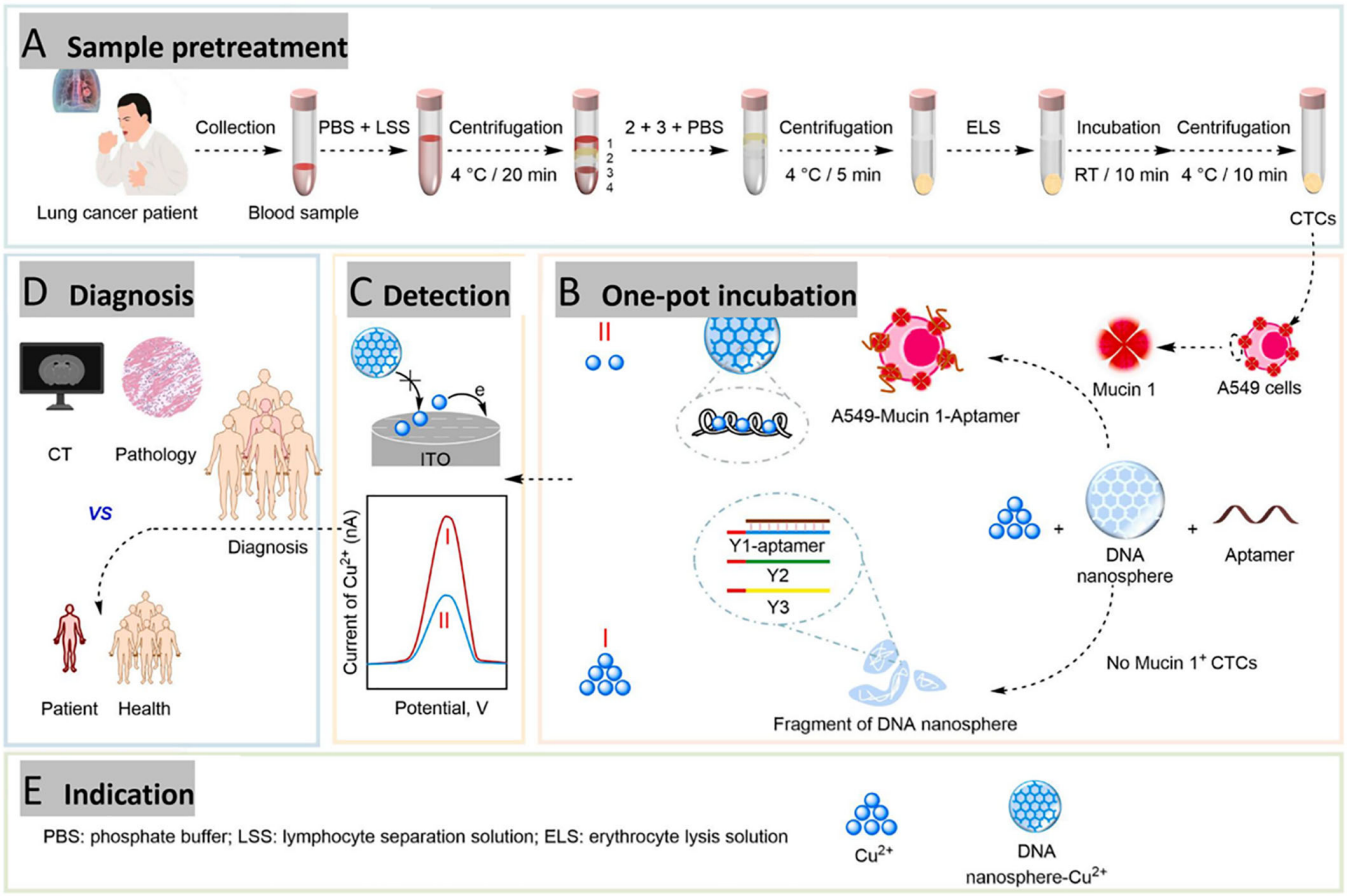
Schematic diagram of the homogeneous electrochemical assay of CTCs in lung cancer patients. Detection process: A) Sample pretreatment. B) One‐pot incubation. C) Detection. D) Diagnosis. E) Indication. Reproduced with permission.^[^ ^214^ ^]^ Copyright 2024, Elsevier.

CTCs detection is important for real‐time monitoring of tumor dynamics, assessment of treatment effects, and prediction of disease metastasis and recurrence. By capturing and detecting CTCs present in peripheral blood, signs of metastasis of tumor cells can be detected early, providing a basis for clinical decision‐making. However, the level of CTCs in blood is extremely low, and the sensitivity and specificity of their detection still face many challenges now. Wu et al. developed a simple and efficient electrochemical method for the detection of CTCs. First, a DNA nanosphere was constructed by hybridization of three single‐stranded DNA sequences (Y1, Y2, Y3). The DNA nanospheres were able to bind to Cu^2^⁺ and significantly reduce the concentration of free Cu^2^⁺, thus enabling quantitative analysis by changes in electrochemical signals. Next, the researchers used lymphocyte isolate and erythrocyte lysate to isolate and enrich CTCs by a three‐step centrifugation operation in 45 min. Then, CTCs were detected based on the specific recognition of mucin 1 and the modulation of Cu^2^⁺ electrochemical signals by DNA nanospheres. The results showed that the detection limit of this method was 2 ag mL^−1^ for mucin 1 and 1 cell mL^−1^ for A549 cells. This method achieves efficient detection of CTCs in lung cancer and has important potential for clinical application (**Figure** **14**).^[^ ^209^ ^]^ The special properties of nanomaterials, such as large specific surface area and high surface energy, are utilized to achieve efficient separation and detection of CTCs.

#### 4.2.2 Efficient Enrichment and Elimination of CTCs

This section can be subdivided into two strategies: 1) Direct targeting and killing of CTCs: by taking advantage of the unique advantages of nanotechnology, NPs or nanocarriers that can specifically recognize and directly kill CTCs are designed and prepared. These nanomaterials deliver anticancer drugs or killing molecules directly into the interior of CTCs through precise targeting, thus achieving efficient killing. 2) Enhancement of indirect killing of CTCs by immune cells: with the help of nanotechnology, we can enhance the efficiency of immune cells in the peripheral blood in recognizing and killing CTCs. By carrying immunostimulants or immunomodulators through NPs, we can activate and enhance the ant‐tumor activity of immune cells, so that they can remove CTCs in the blood more efficiently. These two nanostrategies complement each other, and together they constitute an effective killing system against CTCs, which provides new ideas and methods for the prevention and treatment of blood metastasis of lung cancer.

Huang et al. designed a magnetic MoSe2 nanosheet (PD‐L1‐MFP NS) loaded with PD‐L1.PD‐L1‐MFP NS precisely targets CTCs in the peripheral blood via PD‐L1 and directly kills the CTCs using a highly efficient PTT. At the same time it further activates natural killer cells (NK cells) by increasing the expression of the ul16‐binding protein of the cytomegalovirus on tumor cells. ligand expression and further activates NK cells. Through the dual action of direct killing of CTCs and activation of NK cells, the therapeutic effect can be significantly improved and the risk of lung cancer metastasis can be reduced.^[^ ^210^ ^]^

Compared with single CTCs, circulating tumor cell clusters (CTM) have unique phenotypic and molecular characteristics, with higher metastatic and apoptosis resistance potential. The metastatic risk is 23–50 times higher than that of a single CTC. Therefore, inducing apoptosis of CTM and blocking their extravasation are crucial for inhibiting lung cancer metastasis. Digoxin, as a drug with specific biological activity, was found to have the unique efficacy of dissociating CTM and significantly reducing the extravasation of CTCs. In the field of cancer metastasis research, this discovery undoubtedly provides new ideas and methods to inhibit tumor metastasis. Harada et al., based on this important discovery, innovatively designed and constructed a nano drug delivery system. This system is cleverly loaded with digoxin, which can effectively dissociate CTC clusters by taking advantage of its unique pharmacological effects. By precisely targeting and acting on CTCs, this system not only significantly reduced the vascular extravasation of CTCs, but also effectively blocked the pathway of CTCs spreading to other sites through blood circulation, thus greatly reducing the formation of new metastatic foci, providing a new strategy and hope for the treatment and prevention of metastasis in cancer.^[^ ^211^ ^]^

CTCs are often encapsulated by platelets, neutrophils, or tumor‐derived stromal cells, which can protect them from immune surveillance. Aboul‐Soud et al. synthesized zinc oxide nanoparticles (ZnO‐NPs) using a ZnCl_2_.2H_2_O salt precursor and an aqueous extract of kidney fern (N. exaltata). ZnO‐NPs exhibit potential antiplatelet activity by inhibiting platelet activating factor and arachidonic acid (AA), which induce platelet aggregation. The results showed that ZnO‐NPs effectively inhibited AA‐induced platelet aggregation, were biocompatible, and had significant inhibitory effects on lung cancer growth and metastasis.^[^ ^212^ ^]^

### 4.3 Targeting Metastasized and Colonization Stages

The third stage in the invasion‐metastasis cascade of lung cancer is lung cancer cell colonization and proliferation in distant metastasis and the formation of dormant micrometastasis, followed by the formation of large metastasis at the right time. The “seed and soil” hypothesis was proposed by Paget in 1889, suggesting that tumor cells are the “seeds” of lung cancer metastasis and the target organ is the “soil” in which the seeds grow. Specific tumor cells tend to metastasize to specific target organs. Therefore, both “seed” and “soil” are key factors in the metastasis of lung cancer.

#### 4.3.1 Modulating the Premetastatic Niche

Only fertile “soil” allows “seeds” to thrive. Although CTCs or CTM have great potential for metastasis, their extravasation, proliferation, and colonization in metastatic foci require suitable “soil.” The shaping of the premetastatic niche (PNM) provides a suitable “soil” for the metastasis of tumor cells, which refers to the microenvironment conducive to the growth and proliferation of tumor cells that already exists in the target organ or tissue before the tumor cells reach it. This microenvironment consists of a variety of cellular components (such as stromal cells, immune cells, etc.) and noncellular components (such as ECM, cytokines, etc.).^[^ ^213^, ^214^, ^215^ ^]^ Therefore, disrupting PMN can destroy the “soil” of lung cancer metastasis and prevent the “seeds” from colonizing, which provides a new strategy and direction for suppressing lung cancer metastasis.

**Figure 15:**
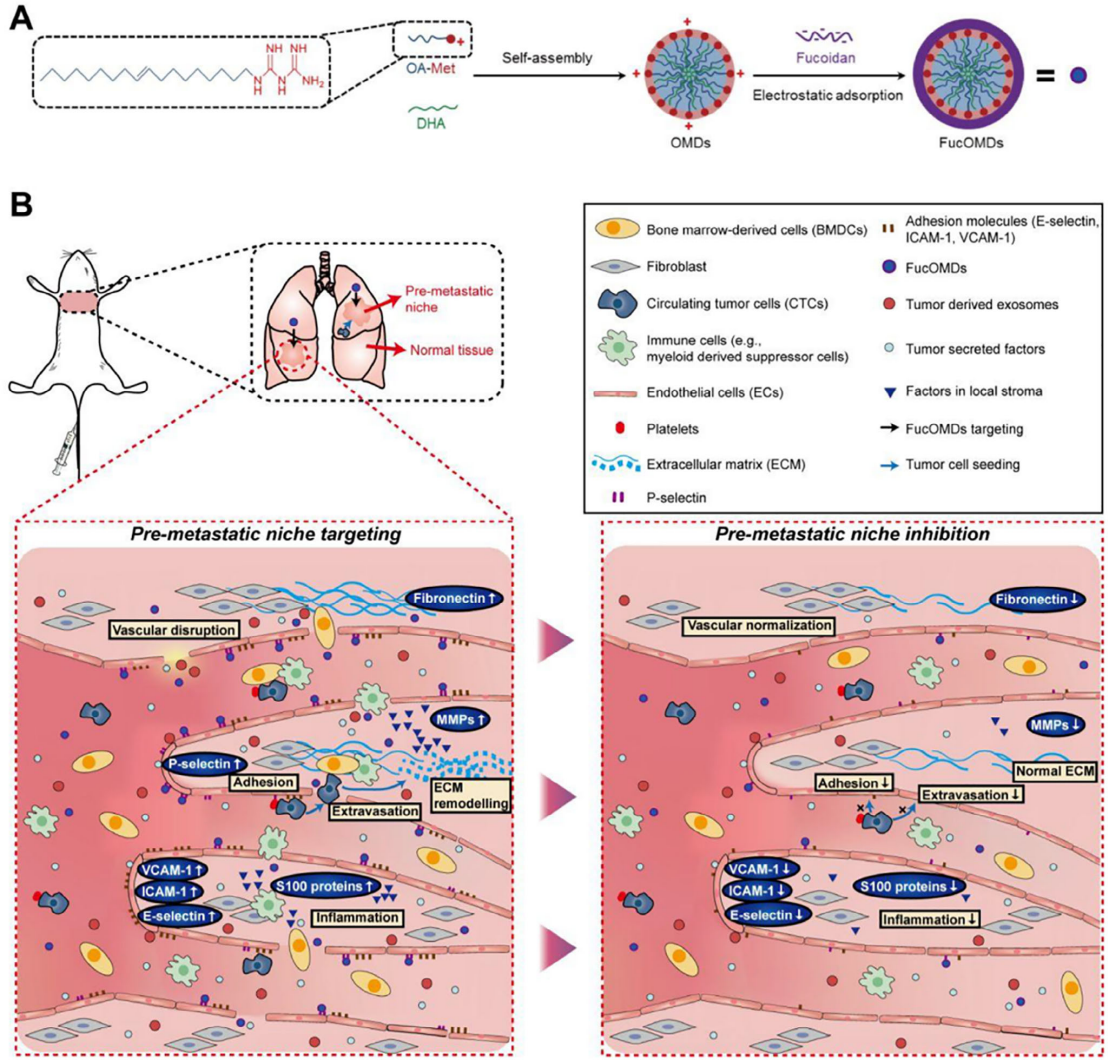
Nanostrategies inhibit lung cancer metastasis through modulating the premetastatic niche. A) Preparation and B) schematic illustration of fucoidan‐decorated self‐assembly micelles delivering metformin and DHA (FucOMDs) for premetastatic niche targeting and modification. Reproduced with permission.^[^ ^221^ ^]^ Copyright 2024, Amer Chemical Soc.

Tianze Jiang developed an NDDS targeting PMN, a novel amphiphilic metformin derivative, and self‐assembled it with docosahexaenoic acid to form micelles (OMDs). To enhance targeting, the surface of the micelles was modified with algal polysaccharides to form functionalized micelles (FucOMDs). Alginate polysaccharides are able to specifically bind to P‐selectin overexpressed in PMN for targeted delivery. It was shown that FucOMDs significantly reduced vascular leakage in the lungs and reversed the aberrant expression of key marker proteins (e.g., fibronectin, MMP‐9, and S100A9) in PMN in a tumor‐conditioned medium‐stimulated mouse model. And when combined with chemotherapeutic agents (CK‐PTX NPs), they significantly inhibited primary tumor growth and metastasis (**Figure** **15**).^[^ ^216^ ^]^

#### 4.3.2 Targeting Disseminated Tumor Cells in Micrometastasis

LCTCs colonize and survive after reaching the target organ and are called disseminated tumor cells (DTCs), which form the “seed” for lung cancer metastasis. Because these surviving “seeds” initially remain in a long‐term dormant state, they are not sensitive to chemotherapy and targeted therapy and can be latent in the patient for a long time. Upon activation, DTCs can cause extensive lung cancer metastasis. Effective therapeutic options and tracking tools for DTCs are currently lacking.^[^ ^217^, ^218^, ^219^ ^]^ Studies have shown that immune escape from dormant DTC is overcome by T‐cell immunotherapy. Focusing on the metastatic TME, Abana et al. investigated the effects of NBTXR3 NPs injected into primary tumors and XRT plus dual‐agent immunotherapy on secondary tumor‐infiltrating T cells in a mouse model of lung cancer. The results showed that NBTXR3 significantly increased the proportion of conserved CDR3b clonotypes in secondary tumors in mice that received only HD XRT for the primary tumor (group 2 vs group 1, *p* = 0.0476). When secondary tumors were exposed to LD XRT, NBTXR3 increased the frequency of overlapping clonotypes, although not significantly (group 4 vs group 3, *p* = 0.3095). Interestingly, there were significantly more CDR3b libraries in secondary tumors shared between Groups 2 and 4 receiving NBTXR3 than in those shared between Groups 1 and 3 (*p* < 0.0001). Comparison of CDR3b normalized Shannon clonality indices and Circos plots did not show any library diversity between the NBTXR3‐treated and non‐NBTXR3‐treated groups. These results confirmed that NBTXR3 nanomedicine injected into primary tumors coupled with XRT plus dual‐agent immunotherapy increased secondary tumor‐infiltrating T cells in a mouse model of lung cancer.^[^ ^220^ ^]^

#### 4.3.3 Eliminating Established Macrometastasis and Preventing Recurrence

Numerous clinical practices have shown that the therapeutic effects of tumor metastasis are poor compared to those of primary foci. This phenomenon involves a complex biological mechanism. 1) Biological differences: malignant evolution of metastasis: metastatic tumor cells are usually screened out from the primary foci as more aggressive and drug‐resistant subclones, and may carry different genetic mutations (e.g., driver mutations, drug‐resistant mutations, etc.) from those in the primary foci, and are therefore insensitive to the original therapeutic agents. In addition, metastatic cells may adapt to the microenvironment of the new organ by adjusting metabolic pathways (e.g., glycolysis, glutamine metabolism), high expression of drug efflux pumps (e.g., P‐glycoprotein), or detoxification enzymes (e.g., glutathione‐S‐transferase), resulting in failure of conventional therapy. 2) The microenvironment of different metastatic sites (e.g., bone, brain, liver, lung, adrenal gland) varies significantly, resulting in variable therapeutic efficacy. Nanotechnology is expected to improve this.

##### Suppression of Drug Efflux Pumps

Patients with EGFR activating mutations are initially effective on tyrosine kinase inhibitor (TKI) therapy, such as gefitinib, but often develop acquired resistance.P‐gp is a membrane transporter protein encoded by the MDR1 gene that is widely expressed in tumor cells. P‐gp is a membrane transporter protein encoded by the MDR1 gene, which is widely expressed in tumor cells, and its main function is to pump a variety of hydrophobic drugs and chemotherapeutic agents out of the cell, thus reducing the intracellular concentration of the drugs and making the cells resistant to the drugs. Li et al. have developed a new type of nano blocking agent to overcome the resistance to the drugs. This nano blocker is ZIF‐8@Gefitinib@HA Nanoparticle (ZIF‐8@G@HA NP). By loading gefitinib onto a zeolite imidazolium ester backbone‐8 (ZIF‐8) nanoplatform and coating it with HA, ZIF‐8 degrades, and releases zinc ions (Zn^2^⁺) in acidic environments, and the HA coating confers the NPs with the ability to bind to the CD44 receptor, facilitating internalization in NSCLC cells. One highlight is that ZIF‐8@G@HA NP can assist in overcoming gefitinib resistance by inhibiting the expression of P‐gp (P‐glycoprotein, a multidrug resistance protein).^[^ ^221^ ^]^

##### Targeting Liver Metastasis of Lung Cancer

**Figure 16:**
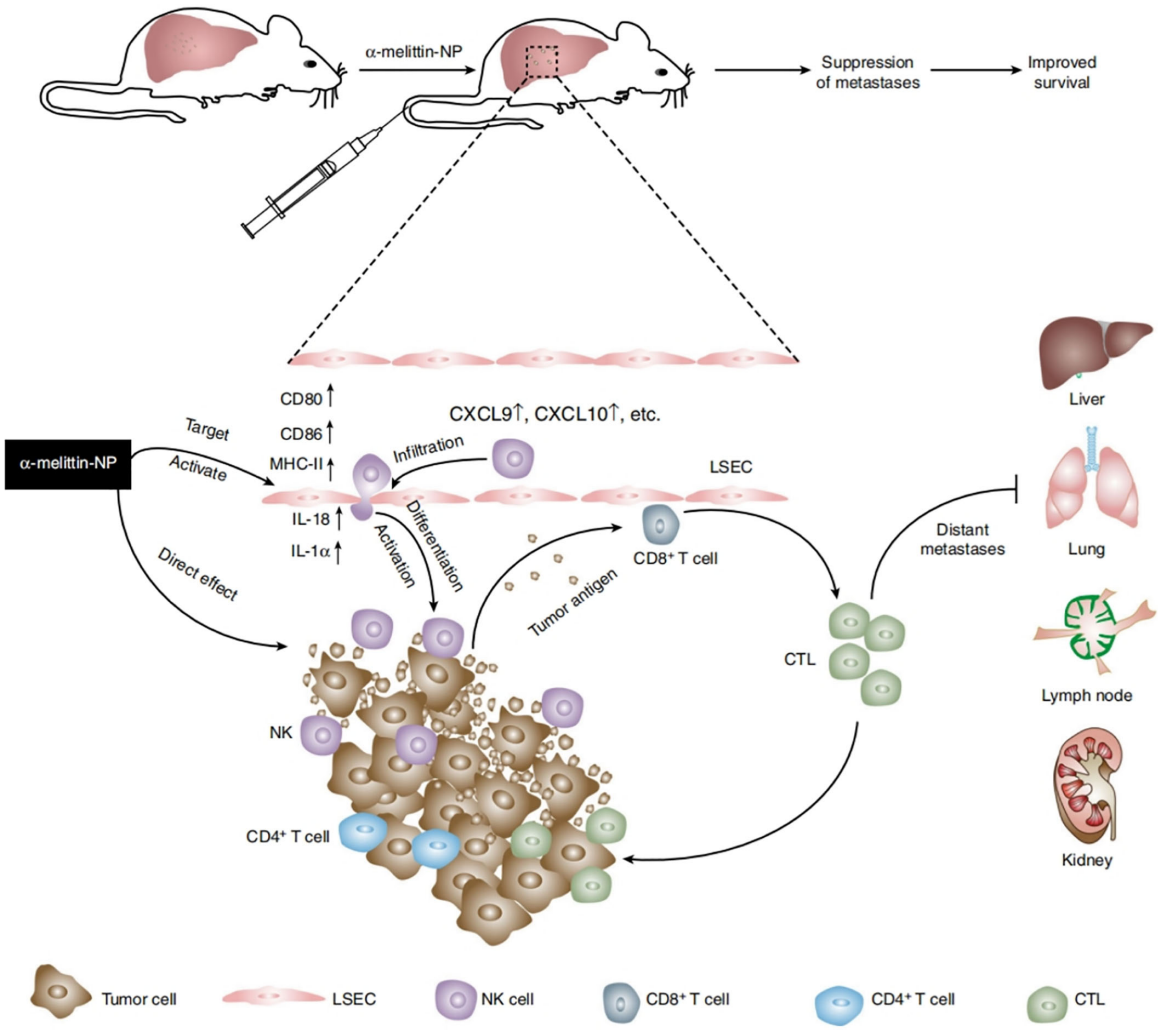
Schematic description of a plausible mechanism for α‐melittin‐NP‐mediated suppression of liver metastasis. α‐melittin‐NPs exert a specific modulatory effect on LSECs, leading to elevated expression levels of costimulatory molecules, such as CD80, CD86, and MHC‐II, as well as the secretion of cytokines and chemokines including IL‐1α, IL‐18, CXCL9, and CXCL10. Upon activation by α‐melittin‐NPs, LSECs trigger the migration of diverse innate and adaptive immune cells to the liver, where they orchestrate protective T‐cell immunity in concert with NK cells, effectively inhibiting liver metastasis. Reproduced with permission.^[^ ^227^ ^]^ Copyright 2019, Nature Publishing Group.

Although chemotherapy, targeted therapy and immunotherapy have achieved remarkable results in the treatment of lung cancer, they are often ineffective in patients with liver metastasis. Chemotherapy and targeted therapy are limited by impaired liver function. The special immune microenvironment of the liver may lead to a discounted effect of immunotherapy. Liver sinusoidal endothelial cells (LSECs), which are the key cells for immune tolerance in the liver, usually exhibit low levels of costimulatory molecule expression and high levels of immunosuppressive factors (e.g., IL‐10 and TGF‐β), resulting in an inability to efficiently activate antitumor immune responses. There is an urgent need to develop a therapy that can specifically target and modulate LSECs to break their immune tolerance and activate antitumor immune responses in the liver, thereby inhibiting the formation of liver metastasis. As shown in **Figure** **16**, Yu et al. designed a type of NPs (α‐melittin‐NPs) consisting of bee venom peptides (melittin) and lipids, with bee venom peptides possessing both immune‐modulatory and cellular toxic effects, while the lipid layer shielded the bee venom peptide from hemolytic toxicity, allowing it to be administered via intravenous injection. It was confirmed that α‐melittin‐NPs could rapidly target LSECs and accumulate in the liver, significantly activating LSECs and altering their immune tolerance phenotype. Specifically, α‐melittin‐NPs enhances the antitumor immune response in the liver by up‐regulating costimulatory molecules and cytokines, recruiting and activating immune cells, such as NK cells and T cells. In multiple tumor models, α‐melittin‐NPs significantly inhibited the formation of liver metastasis and prolonged the survival of mice, especially in the spontaneous liver metastasis model, with a survival rate of 80%. Overall, this study demonstrates the great potential of nanotechnology in inhibiting lung cancer liver metastasis and provides a new direction for the treatment of lung cancer liver metastasis.^[^ ^222^ ^]^

##### Targeting Brain Metastasis of Lung Cancer

**Figure 17:**
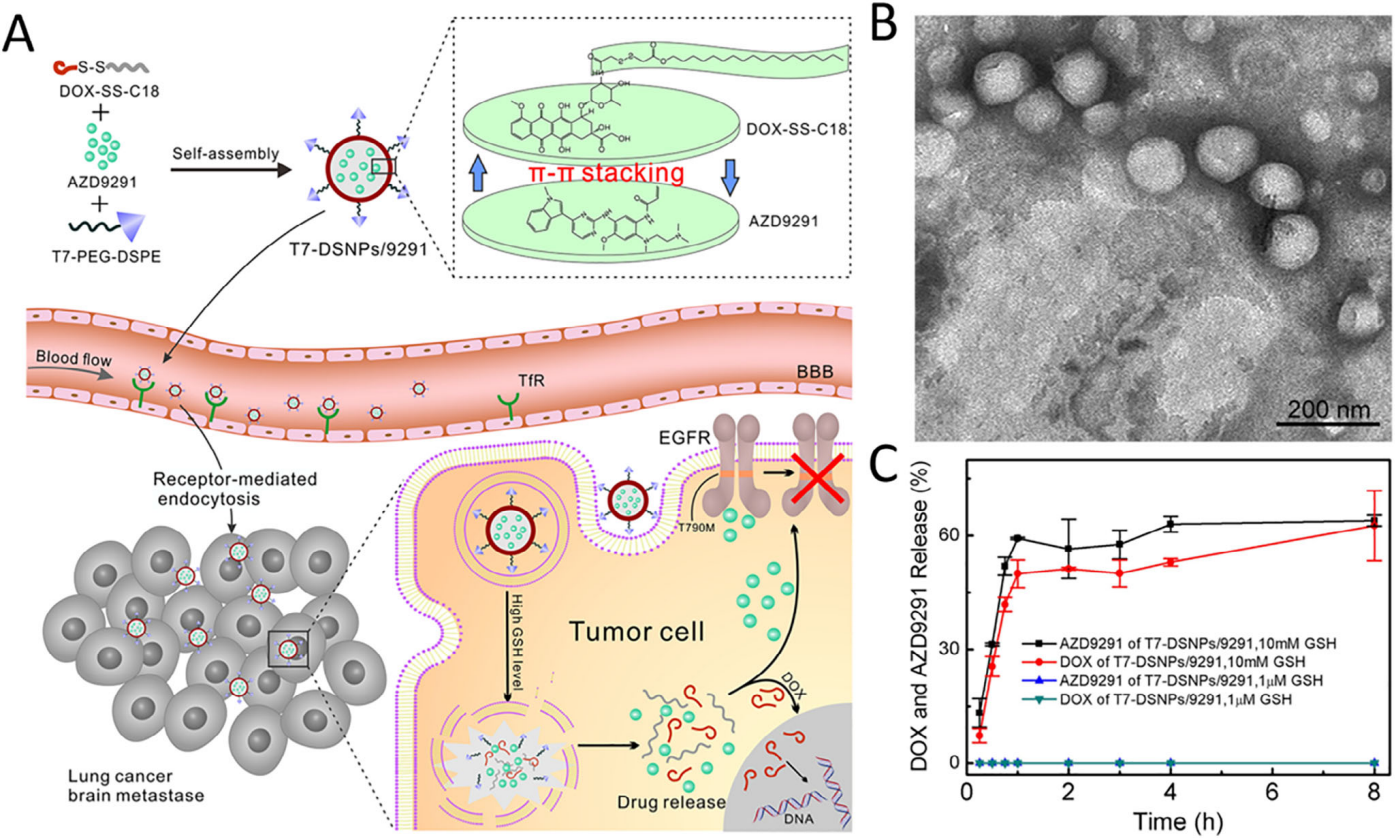
Applications of nanomedicine in multidrug codelivery. A) Preparation and schematic representation of nanomedicine T7‐DSNPs/9291 loaded with both ositinib (AZD9291) and doxorubicin. B) Transmission electron microscopy images of T7‐DSNPs/9291. C) Reduction‐triggered release of payload from T7‐DSNPs/9291 in different release condition (*n* = 3). Reproduced with permission.^[^ ^228^ ^]^ Copyright 2020, Dove Medical Press Ltd.

Treatment of brain metastasis from lung cancer is clinically very difficult. The presence of the blood–brain barrier greatly restricts the penetration of drugs into brain tissues, making the treatment of lung cancer brain metastasis extremely tricky and challenging in the clinic. Wang et al. developed a NDDS capable of penetrating the blood–brain barrier (BBB) (**Figure** **17**). It is a self‐assembled nanocarrier based on GSH‐responsive DOX prodrug (DOX‐SS‐C18) loaded with ositinib (AZD9291). To enhance the BBB penetration of the nanocarriers, the surface was modified with a T7 peptide (CHAIYPRH), which was able to enhance the BBB penetration of the nanocarriers via transferrin receptor (TfR)‐mediated transcytosis. BBB penetration experiments demonstrated that the T7‐DSNPs/9291 was able to significantly enhance the ability of both AZD9291 and DOX to cross the BBB, and that the process was dependent on the interaction of T7 peptide with TfR. Real‐time fluorescence imaging experiments showed that the brain accumulation of T7‐DSNPs/9291 was significantly higher than that of the unmodified nanocarriers in an intracranial PC‐9 tumor mouse model, suggesting a good BBB penetration ability. Overall, T7‐DSNPs/9291, as a brain‐targeted nanocarrier, was able to effectively deliver AZD9291 and DOX to brain metastatic lesions, significantly enhancing the therapeutic efficacy against NSCLC brain metastasis. This study provides a potential drug delivery strategy for the combination therapy of NSCLC brain metastasis.^[^ ^223^ ^]^

**Figure 18:**
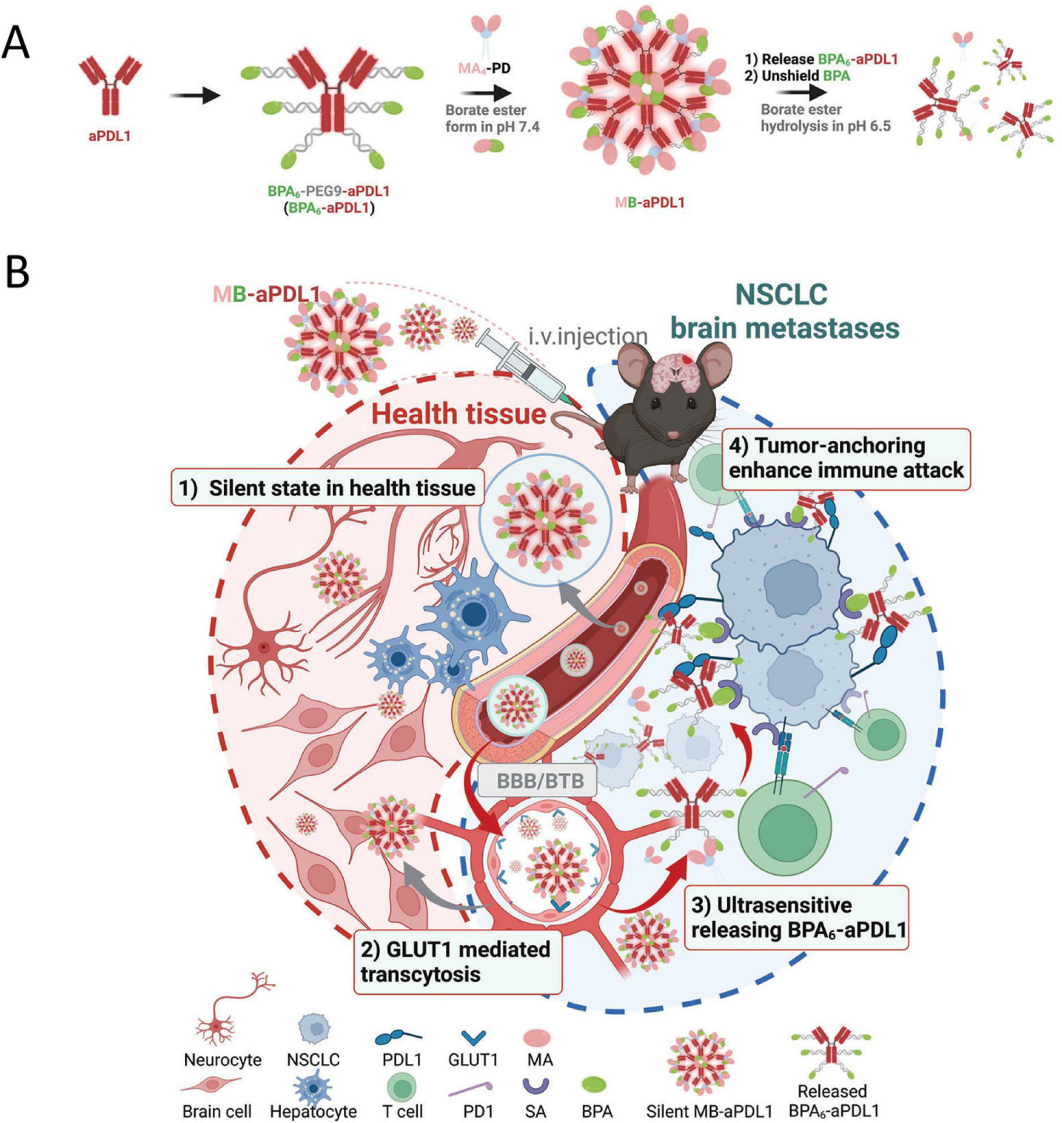
Nanostrategies inhibit lung cancer metastasis through eliminating established macrometastasis and preventing recurrence. A) Scheme of MB‐aPDL1 synthesis and borate ester form in pH 7.4 and switch to hydrolyze in pH 6.5. B) Schematic illustration of the multistage‐responsive antibody‐delivery system MB‐aPDL1 crossing BBB/BTB to treat NSCLC brain metastasis, remain inactive in healthy tissues with reduced immune‐related adverse events, and promote ultrasensitive release of BPA6‐aPDL1 that anchors specifically PD‐L1‐positive tumor cells. Reproduced with permission.^[^ ^229^ ^]^ Copyright 2024, Wiley‐VCH.

Immune checkpoint blockade therapy has shown great potential in clinical oncology, but the treatment of brain metastasis is challenged by the BBB and immune‐related adverse events. Li et al. developed a multistage responsive antibody delivery system that penetrates the BBB, precisely releases antibodies in the TME and reduces immune‐related adverse events. This is an antibody delivery nanoformulation (MB‐aPDL1) based on an ultra‐pH‐sensitive boronic acid bond, which is capable of maintaining a “silent state” in healthy tissues, penetrating the BBB/BTB through GLUT1‐mediated transcytosis, and responsively releasing functionalized aPDL1 (anti‐PD‐L1 antibody) in the TME to promote aPD‐L1 antibody delivery and reduce the number of irAEs. Real‐time fluorescence imaging experiments confirmed that the brain accumulation of MB‐aPDL1 was significantly higher than that of unmodified aPDL1 in a mouse model of intracranial NSCLC brain metastasis, suggesting a good BBB penetration ability. Assessment of the tumor suppression ability revealed that MB‐aPDL1 significantly inhibited intracranial NSCLC tumor growth and prolonged the survival of mice with a cure rate of 20% (**Figure** **18**).^[^ ^224^ ^]^

##### Targeting Bone Metastasis of Lung Cancer

**Figure 19:**
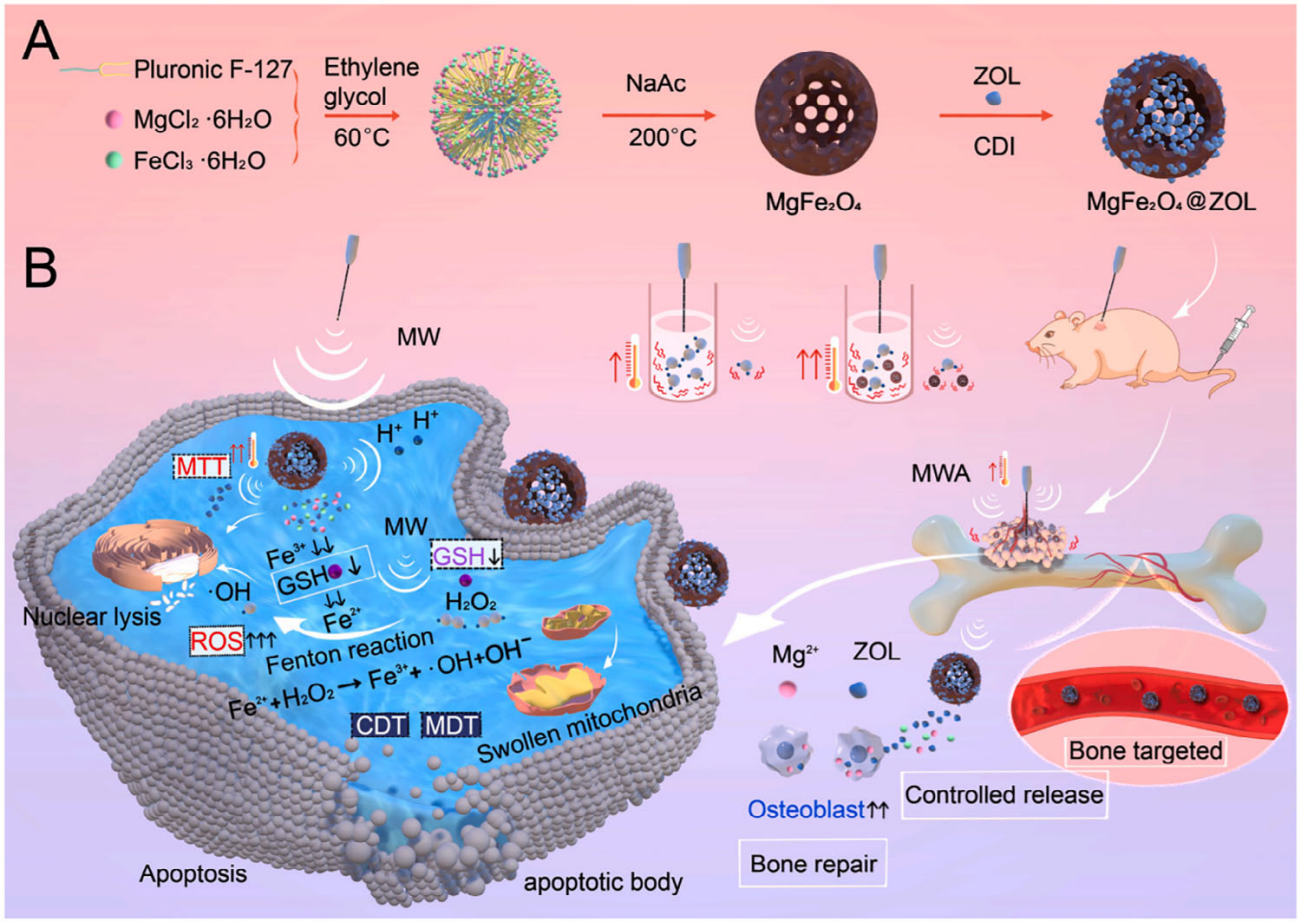
Schematic illustration of the microwave and MgFe2O4@ZOL NPs used in synergistic tumor therapy. A) The synthesis process of MgFe2O4@ZOL NPs. B) Schematic diagram of synergistic therapy involving targeting, CDT, MDT, and selective‐MTT, and bone repair for bone metastasis. Reproduced with permission.^[^ ^230^ ^]^ Copyright 2024, Elsevier.

About 30–40% of lung cancer patients will develop bone metastasis. Bone metastasis lead to bone destruction, which seriously affects patients' quality of life. Current mainstream therapies mainly focus on inhibiting the growth of cancer cells and preventing bone destruction, but suffer from low therapeutic efficiency and high side effects. Shu et al. developed a novel therapeutic approach combining nanotechnology and microwave ablation to improve the therapeutic efficacy of bone metastasis in lung cancer. The team constructed a microwave‐responsive nanoplatform, MgFe₂O₄@ZOL, which consists of spinel‐type magnesium ferrite (MgFe₂O₄) NPs and zoledronic acid (ZOL). The MgFe₂O₄ NPs were synthesized by solvothermal method and have a hollow mesoporous structure, which can be efficiently loaded with ZOL. This study verified the microwave heating efficiency, drug release behavior, targeting, ROS generating ability, and killing effect of MgFe₂O₄@ZOL NPs on cancer cells by in vitro and in vivo experiments. It was confirmed that MgFe₂O₄@ZOL NPs could significantly enhance the effects of chemodynamic therapy (CDT) and microwave kinetic therapy (MDT) under microwave irradiation, effectively inhibit the growth of bone metastasis of lung cancer, and promote bone repair. The MgFe₂O₄@ZOL NPs were evaluated by cytotoxicity assay and hemolysis assay, which showed that the MgFe₂O₄@ZOL NPs had less toxicity to normal cells in vitro and did not cause significant hemolysis reaction. This study provides new ideas and methods for the treatment of lung cancer bone metastasis, and has important clinical translational value (**Figure** **19**).^[^ ^225^ ^]^

##### Targeting Lung Metastasis of Lung Cancer

CpG oligodeoxynucleotides are potent Toll‐like receptor (TLR) 9 agonists that have shown promise as anticancer agents in preclinical studies and clinical trials. The binding of CpG to TLR9 triggers a series of innate and adaptive immune responses, beginning with the activation of dendritic cells and leading to a series of secondary effects, including the secretion of proinflammatory cytokines, activation of natural killer cells, and T‐cell expansion. Compared with systemic administration, local delivery of CpG in tumors leads to superior antitumor effects. Perry et al. utilized PRINT (particle replication in nonwetting templates) NPs as a vehicle to deliver CpG into murine lungs via orotracheal drip. In two murine in situ metastasis models of NSCLC‐344SQ (lung adenocarcinoma) and KAL‐LN2E1 (lung squamous carcinoma), the local delivery of PRINT‐CpG to the lungs effectively promoted substantial tumor regression and limited the systemic toxicity associated with soluble CpG. In addition, the cured mice were completely resistant to tumor re‐attacks. In addition, nano drug delivery showed prolonged retention of CpG in the lungs and prolonged elevation of antitumor cytokines in the lungs, with no increase in serum proinflammatory cytokine levels. These results suggest that the TLR 9 agonist CpG oligonucleotide, which is delivered by pulmonary administration using NPs as a carrier, could be used for the treatment of lung cancer metastasis or other metastatic lung cancers locally.^[^ ^226^ ^]^

##### Targeting Adrenal Metastasis of Lung Cancer

There are fewer reports of nanostudies targeting adrenal metastasis from lung cancer, but nanotechnology has broad applicability in adrenal metastasis prevention. For example, the development of adrenal‐specific targeted nanocarriers, and exploring the combination of local delivery (e.g., percutaneous intervention) and systemic therapy.

## 5 Challenges in Clinical Translation

Nanomedicine technology has evolved over the past two decades and a multitude of nanomedicines have been approved for clinical use (**Table** **4**). Various nanomedicines are in the clinical trial stage (**Table** **5**). However, as seen from the results of clinical trials, the clinical translation efficiency of nanomedicines is still very low and is affected by some factors. To summarize the development of nanomedicines that are in the clinical trial stage in recent years.1) Liposomes: mature process, low toxicity, but ABC effect, poor stability; 2) Polymer micelles: no solvent required, low cost, but inflammation risk, and limited drug loading; 3) Nucleic acids: precise gene regulation, individualized therapy, liver targeting, but harsh storage conditions; 4) Inorganic nanoparticles: high stability, diagnosis, and treatment integration, but easy to accumulate toxicity and dependent on external equipment; 5) exosomes: natural targeting, low immunogenicity, but complex production and low drug loading efficiency.^[^ ^227^, ^228^, ^229^, ^230^ ^]^ Currently, liposomes are still the mainstream, accounting for about 40%, but stability and immune clearance issues need to be addressed. Nucleic acid drugs are emerging, such as mRNA vaccines and CRISPR vectors, but delivery efficiency is still a bottleneck.

**Table 4:** Four nanomedicines approved globally for lung cancer.

Drugs	Company	Nano types	Date of approval	Advantages
Abraxane	Celgene (US)/BeOne Medicines Ltd. (China)	Paclitaxel Albumin Nanoparticles	FDA: 2012 NMPA: 2013	Targeting SPARC proteins in the tumor microenvironment using albumin to reduce allergic reactions and increase intra‐tumor drug concentrations.
Lipusu	CSPC Pharmaceutical Group Limited (China)	Docetaxel Albumin Nanoparticles	NMPA: 2022	No polysorbate solvent is required, reducing infusion reactions and improving patient tolerance.
Genexol‐PM	Samyang Biopharm (South Korea)	Paclitaxel polymer micelles	MFDS: 2007	Solvent‐free formulation that targets tumors through enhanced permeation retention (EPR) effect.
Paclitaxel polymer micelles for injection	Shanghai Yizhong Pharmaceutical Co., Ltd. (China)	Paclitaxel micelles	NMPA:2021	No preantiallergic treatment is required, making clinical use more convenient.

**Table 5:** Global clinical trials related to lung cancer nanotherapies.

Nanocarrier types	Clinical trials	Phase	No.	Result
Liposomes and derivatives	Onivyde in Combination with Keytruda	II	NCT04028430	In progress.
	ThermoDox Combined Radiofrequency Ablation	II	NCT02379858	Has been terminated. Terminated due to lack of efficacy, but validated the feasibility of heat‐triggered drug release.
	Lipoplatin versus conventional cisplatin	III	NCT00355888	Has been terminated. Median survival was extended by 3 months and nephrotoxicity was reduced by 50%.
Polymer nanoparticles	Paclitaxel micelles (Shanghai Yizhong Pharmaceutical Co., Ltd.)	III	NCT04338399	Has been terminated. ORR of 50% without antiallergic pretreatment.
	Genexol‐PM in combination with cisplatin	III	NCT03326193	In progress.
	BIND‐014	II	NCT01283334	Has been terminated. Terminated due to lack of efficacy, but technology acquired by Novartis for optimization.
Nucleic acid nanomedicines	BNT111 (individualized neoantigen vaccine) in combination with a PD‐1 inhibitor	II	NCT04503278	In progress.
	siRNA targeting of KRAS G12C mutation (AZD4785)	I	NCT03419572	Has been terminated. Terminated due to insufficient delivery efficiency, but provides data for subsequent LNP optimization.
Inorganic nanoparticles	NBTXR3 (radiotherapy sensitizer)	I/II	NCT04784221	In progress.
	Aurolase (photothermal therapy)	I	NCT03817411	In progress.
Other new types	ExoASO‐STAT6 (exosome‐carrying STAT6 antisense oligonucleotide)	I	NCT05232812	In progress.

In addition, there are a series of bottlenecks and challenges in the clinical translation of nanomedicines. First, nanomedicines must undergo a complex multistep cascade in vivo to be effective, including injection into the blood circulation, accumulation at the tumor site, penetration into the interior of the tumor tissue, endocytosis, intracellular transport, and drug release. The complexity of the environment of the human body affects the distribution and metabolism of nanomaterials. Uncertainties and variables in this process exist, and their safety is yet to be verified, making the development and translation of nanomedicines challenging. Second, nanobiological interactions are important considerations. The TME is a complex system which includes tumor cells, vascular endothelial cells, tumor‐associated fibroblasts, and immune cells, as well as pathophysiological features, such as hypoxia and acidosis. The efficacy of nanomedicines mainly depends on their behavior in the TME, including transport into and out of the blood vessels, endocytosis, and accumulation in tumor tissues. These complexities make the interaction between nanomedicines and tumors unpredictable, thereby affecting their efficacy. The current assessment of the efficacy of nanomedicines lacks precision. The models commonly used to assess the efficacy of nanomedicines, such as cell lines and mouse models, do not accurately reflect the heterogeneity of human tumors and the TME. This may contribute to effective outcomes in preclinical settings and poor outcomes in clinical trials. However, research teams are already focusing on the development of organoid models that are expected to play a role in the assessment of nanomedicine efficacy.^[^ ^231^ ^]^ In addition, the scaled‐up effect and complexity of quality control during the translation process are important constraints in the clinical translation of nanotechnology. In the preparation of nanomedicines, it is necessary to scale‐up and ensure the consistency and stability of product quality. This requires in‐depth research and optimization of the production processes, equipment, and quality control. In addition, the clinical transformation of nanotechnology is affected by many aspects, such as research investment, risk awareness, market service awareness, research layout and planning, maturity of scientific and technological achievements, transformation, information communication, industry‐university‐research cooperation, talent cultivation, intellectual property rights protection, and industry standards. Together, these factors constitute a complex environment for the clinical transformation of nanotechnology, which needs to be comprehensively considered and resolved.

## 6 Summary and Perspective

Lung cancer is a malignant tumor with the highest mortality rate. The high mortality rate is primarily attributed to lung cancer metastasis. The MDT model has shown encouraging results in the treatment of lung cancer metastasis in clinical practice. By pooling the wisdom and experience of multidisciplinary experts, this model provides a more personalized, comprehensive and precise treatment plan for patients with lung cancer metastasis. However, although the MDT model has shown significant advantages in the treatment of lung cancer metastasis, it is undeniable that these therapies have encountered bottlenecks and challenges that need to be further explored and solved. With the rapid advancement of nanotechnology, its applications in the medical field are expanding. Various types of nanomaterials, including lipid‐based nanomaterials, polymeric nanomaterials, and MOFs, are being developed to meet increasingly diverse and demanding design needs.^[^ ^232^ ^]^ Delivery systems constructed from these nanomaterials have demonstrated precise targeting, environmentally responsive release, multidrug synergistic delivery, diagnostic and therapeutic integration, innovative routes of administration, excellent biosafety, and good blood stability, which have opened up unprecedented opportunities for innovation and development in medicine. This paper focuses on the recent advances in the field of nanotechnology in the prevention of lung cancer metastasis. Although nanotechnology has made considerable progress in the prevention and treatment of lung cancer metastasis, there are still many serious challenges for future clinical translation. However, it is believed that continued interdisciplinary collaboration, innovative research and technological advances will surely facilitate the development of novel antimetastatic nanomedicines against lung cancer and achieve their effective clinical translation.

## Conflict of Interest

The authors declare no conflict of interest.
